# Development of a metabolome-based respiratory infection prognostic during COVID-19 arrival

**DOI:** 10.1128/mbio.03343-23

**Published:** 2024-11-22

**Authors:** John I. Robinson, Laura R. Marks, Andrew L. Hinton, Jane A. O'Halloran, Charles W. Goss, Peter J. Mucha, Jeffrey P. Henderson

**Affiliations:** 1 Division of Infectious Diseases, Department of Internal Medicine, Washington University School of Medicine, St. Louis, Missouri, USA; 2 Curriculum in Bioinformatics and Computational Biology, University of North Carolina, Chapel Hill, North Carolina, USA; 3 Division of Biostatistics, Washington University School of Medicine, St. Louis, Missouri, USA; 4 Department of Mathematics, Dartmouth College, Hanover, New Hampshire, USA; Rutgers The State University of New Jersey, Piscataway, New Jersey, USA

**Keywords:** COVID-19, metabolomics, prognostic indicators

## Abstract

In a new respiratory virus pandemic, optimizing allocation of scarce medical resources becomes an urgent challenge. Infection prognosis takes on particular importance when allocating scarce antiviral antibodies and drugs, which are most effective when administered before the onset of severe disease. During arrival of the COVID-19 pandemic to the United States in 2020, we conducted a prognostic biomarker discovery and validation effort based upon metabolomic profiling with a liquid-chromatography-mass spectrometer (LC-MS) type used clinically for rapid toxicology. We obtained urine specimens from 163 patients presenting for evaluation. We obtained LC-MS profiles in the initial cohort and used machine learning methods to define a simplified urine metabolomic signature associated with respiratory failure or death by 90 days. This signature was composed of three metabotypes linked to intestinal microbiome metabolism and anticonvulsant use, with a receiver-operator characteristic area under the curve (ROC AUC) of 89.4%. Blinded application of this signature to the subsequent validation cohort yielded a ROC AUC of 81.2%. A model trained on the two baseline metabotypes present before intubation exhibited similar performance in the validation cohort. This study demonstrates the plausibility and promise of rapid metabolome-based prognostic discovery and validation in the opening wave of a pandemic. The approach used here could be used to inform therapeutic and resource allocation decisions early in a future epidemic.

**IMPORTANCE:**

In a new respiratory virus pandemic, the ability to identify patients at greatest risk for severe disease is essential to direct scarce medical resources to those most likely to benefit from them. Tools to predict disease severity are best developed early in a pandemic, but laboratory-based resources to develop these may be limited by available technology and by infection precautions. Here, we show that an accessible metabolic profiling approach could identify a prognostic signature of severe disease in the initial wave of COVID-19, when patients presenting for care often exceeded the available doses of convalescent plasma and remdesivir. In a future pandemic, this approach, alongside efforts to identify clinical disease severity predictors, could improve patient outcomes and facilitate therapeutic trials by identifying individuals at high risk for severe disease.

## INTRODUCTION

The arrival of SARS-CoV-2 over a short interval from late 2019 to early 2020 overwhelmed medical care providers worldwide. The rapid development of SARS-CoV-2 assays was a key element of the pandemic response, first for limiting transmission and soon after for identifying patients that could benefit from convalescent plasma and remdesivir therapy. Optimal allocation of these initially scarce agents required physicians to decide which patients were most likely to benefit. Estimating patient risk was further pressured by the need to administer antiviral agents early, often before respiratory failure was evident (1–6), as is typical of respiratory virus infections (7). As we endeavor to minimize morbidity and mortality in the next pandemic, rapidly ascertaining disease risk is an important area of emphasis.

Early in the COVID-19 pandemic, disease progression risk understandably relied upon readily accessible baseline clinical characteristics such as age, sex, or diabetes diagnosis (8). A recent retrospective chart review of 969 inpatients during the early pandemic indicates that further prognostic information was accessible from additional clinical data beyond baseline clinical characteristics (9). New laboratory biomarkers of risk also have potential to improve prognostic accuracy, but with a new disease, it is difficult to ascertain which of the thousands of possible analytes are most useful. One potential source of risk-associated laboratory biomarkers is the metabolome, the population of small molecules whose presence and abundance in patients reflect a broad array of physiological processes and exposures (10). Of note, clinical and research laboratories currently have the potential to expeditiously identify and deploy metabolome-derived prognostic signatures using the same liquid chromatography-mass spectrometry (LC-MS) profiling approaches currently applied to identify inborn errors of metabolism or drug and toxin exposures. These resources could be useful early in an epidemic when resource limitations imposed by geography and infectious agent precautions (specimen shipping, phlebotomist exposure, etc.) necessitate on-site discovery and validation.

In this study, we used LC-MS to identify metabolomic correlates of severe COVID-19 progression in patients presenting for evaluation at a U.S. academic medical center during local SARS-CoV-2 arrival, prior to vaccine availability. We obtained patient urine specimens upon emergency department admission and assessed their temporal association with severity-defining endpoints of respiratory failure or death. A metabolomic signature associated with severe COVID-19 progression in subjects enrolled early in the study was validated via blinded analysis in later enrollees. The severity-associated signature is composed of three metabotypes, two of which preceded onset of severe disease and one that preferentially appeared after intubation. This prototype assay exhibits a stronger prognostic potential for severe outcomes than a panel of clinical risk factors.

## RESULTS

### Clinical outcomes and SARS-CoV-2+ cohorts

**Fig 1:**
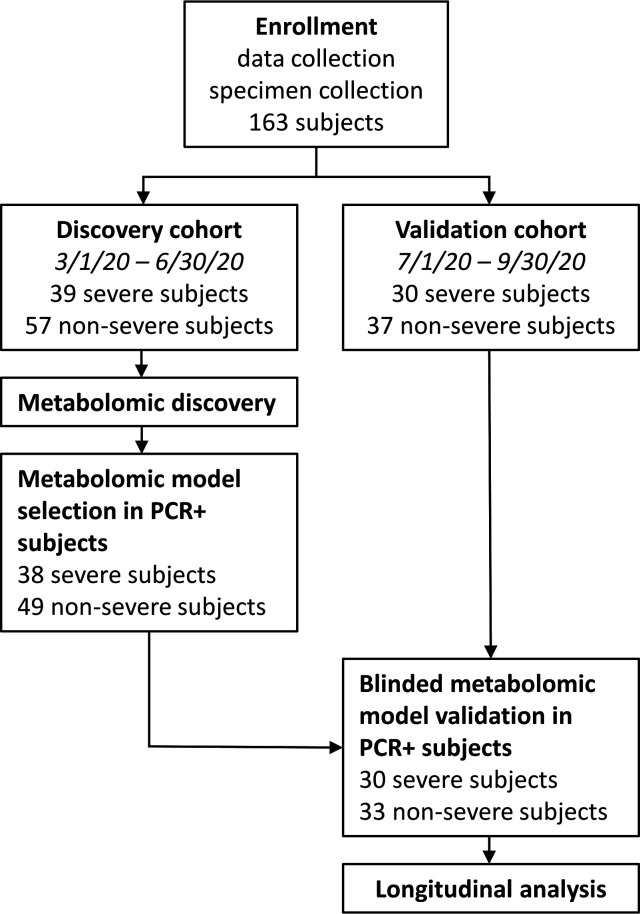
Study design and enrollment.

Between 1 March 2020 and 30 September 2020, 163 patients presenting to the Barnes-Jewish Hospital emergency department with symptoms compatible with COVID-19 or at high risk of developing COVID-19 were enrolled (Fig. 1). Among the 150 SARS-CoV-2-positive patients in our cohorts, 45% (68) reached the pre-defined, severe COVID-19 composite endpoint of intubation or death by 90 days from admission, with all but 2 within 30 days. Of these, 82% (56) was intubated, with 50% (11) mortality post-intubation. Of the 40 COVID-19 patients who died during the study period, death was attributed to COVID-19 in 88%. Cause of death was not available for two COVID-19 patients. Patients with severe COVID-19 were significantly older (median 68.4 vs. 61.2 years, *P* < 0.001) with non-significantly increased diagnoses of diabetes or chronic kidney disease (36.8% vs. 25.6% and 7.4% vs. 2.4%, respectively; Table 1).

**TABLE 1:** SARS-CoV-2 PCR-positive patient characteristics

	Non-severe COVID-19 *N* = 82	Severe COVID-19 *N* = 68	*P* value^*a*^
Demographics			
Male	51 (62.2%)	42 (61.8%)	>0.99
Median age (interquartile range)	61.2 (43.0–69.3)	68.4 (59.9–76.0)	**<0.001** ^*b*^
African American	59 (71.1%)	45 (66.2%)	0.48
Discovery cohort	49 (59.8%)	38 (55.9%)	0.74
Patient comorbidities			
Malignancy or solid organ tumor	4 (4.88%)	2 (2.94%)	0.69
Diabetes mellitus	21 (25.6%)	25 (36.8%)	0.16
Chronic kidney disease	2 (2.44%)	5 (7.35%)	0.25
Asthma or chronic obstructive pulmonary disease	15 (18.3%)	8 (11.8%)	0.36
Congestive heart failure	10 (12.2%)	4 (5.88%)	0.26
COVID-19 outcomes			
Respiratory failure during index hospitalization	0 (0%)	56 (82.4%)	
Death due to COVID-19	0 (0%)	35 (51.5%)^*c*^	

^*a*^ Fisher’s exact test, except where indicated.

^*b*^ Wilcoxon rank-sum test (boldface type indicates statistical significance).

^*c*^ Attribution of cause of death was not available for two deceased PCR-positive patients.

Discovery cohort enrollment ended after 4 months (1 March 2020 to 30 June 2020) with 96 patients. Subsequently, 67 patients were recruited (1 July 2020 to 30 September 2020) to the validation cohort. No significant differences in baseline clinical characteristics were observed between cohorts. When 90-day endpoints were known, we divided each cohort into severe and non-severe patients (39 and 57 for discovery, 30 and 37 for validation). Among severe COVID-19 patients, 57% (39/68) was intubated before urine collection while 50% of their urine specimens was collected between 2.9 days before and 3.5 days after reaching the severity-defining endpoint. Restricting to patients with positive SARS-CoV-2 PCR tests later reduced the discovery cohort to 87 patients (38 severe, 49 non-severe) and the validation cohort to 63 patients (30 severe, 33 non-severe).

### Metabolomic correlates of progression to severe COVID-19

To identify metabolomic variation associated with progression to severe COVID-19, we obtained full-scan LC-MS profiles of the 96 discovery cohort samples (including PCR-negative samples) in positive- and negative-ion modes. Both unsupervised hierarchical clustering and principal component analysis (PCA) of low-sparsity metabolite logratios could partially discriminate severe and non-severe COVID-19 patient groups (Fig. S17 and S18). To better identify correlates of progression to severe COVID-19, we next applied multiple feature filtering and bounded feature selection techniques (see Materials and Methods). Models combining both logratios and clinical characteristics generally outperformed models trained with either data type alone (Fig. S15). Models including positive ion and/or negative ion-derived logratios performed comparably. Using positive-ion mode logratios and clinical characteristics with a maximum of 70 metabolites in our bounded feature selection yielded models with a mean area under the curve (AUC) of 0.801 from, on average, 20.6 metabolites. The Hamming distance between optimized feature subsets defined a single most representative set of 24 metabolites and 4 clinical variables (age, presence of stroke/dementia/seizure disorder, confusion, and fatigue) for further biomarker development. When sample collection for the discovery cohort began, patients with both confirmed and suspected SARS-CoV-2 infection were enrolled due to limitations in SARS-CoV-2 diagnostic performance and availability. Early steps of feature selection were performed on this combined discovery cohort, which contained nine PCR-negative patients (with one severe outcome). We retained these PCR-negative patients to prevent outcome information from biasing our already-performed bounded feature selection. Subsequent LC-MS/MS detection and classifier training (below) on the discovery cohort utilized only the 87 PCR-positive discovery cohort patients.

### Defining a signature of progression to severe COVID-19

We next sought to identify a simplified metabolomic signature of progression to severe COVID-19. To quantify the 24 candidate metabolites with greater sensitivity, specificity, and precision than the full-scan LC-MS mode used for metabolomic discovery, we re-measured each using LC-MS/MS detection (Table S2). Of the 24 metabolites, 5 (21%) were detected in all specimens and 16 (67%) were detected in more than half. As detailed above, we used the DiCoVarML R package (12, 13) to train a model in terms of logratios between five metabolites, yielding the highest average training set AUCs among models trained with four to eight metabolites (Fig. S16).

**Fig 2:**
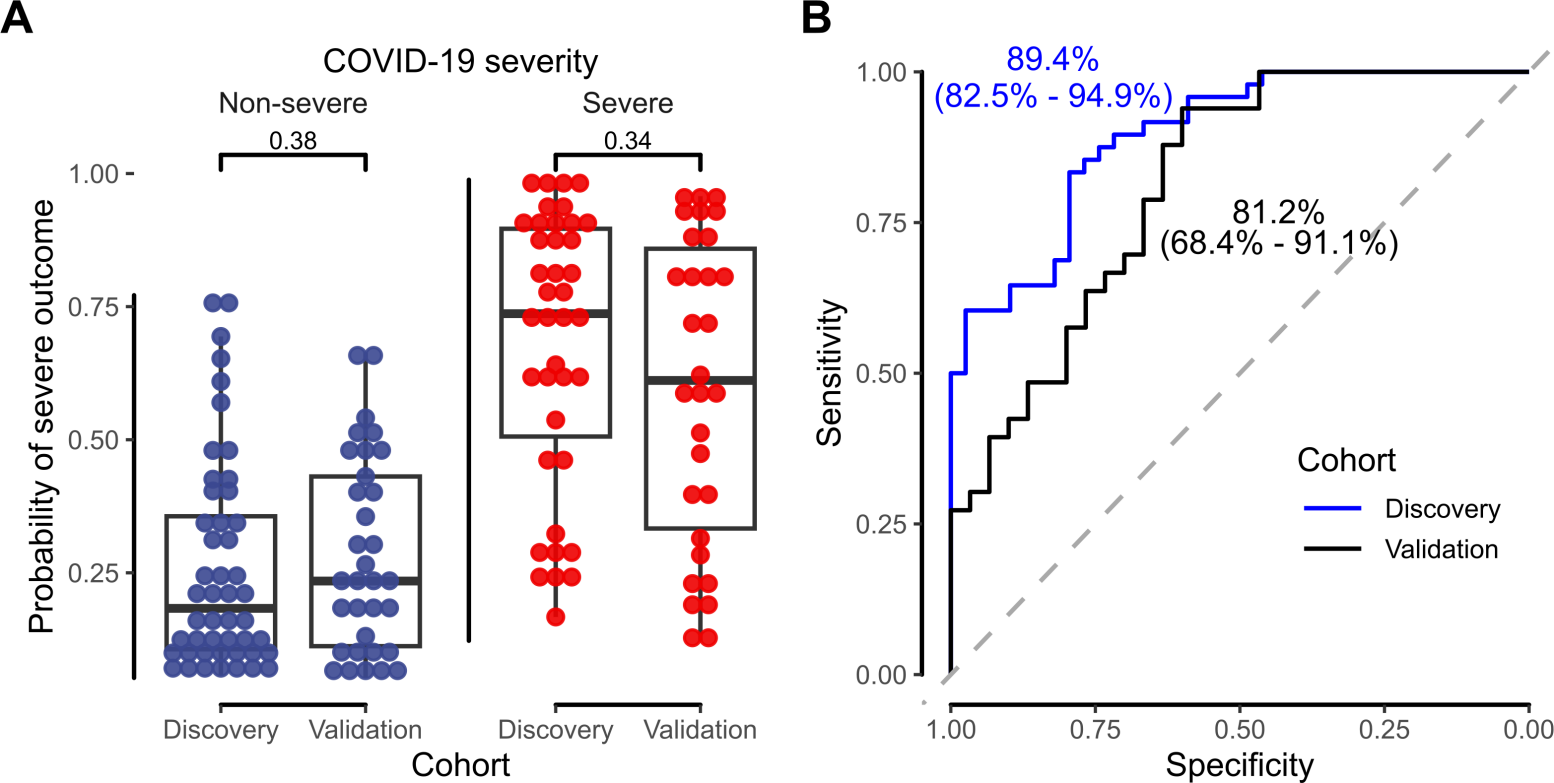
Logistic regression model on a subset of feature logratios confirms association between metabolite logratio signature and COVID-19 disease severity. (**A**) Dot plots of final-model-predicted probabilities that patients reached the severe endpoint, comparing probability distributions between the discovery and validation cohorts (two-sample Kolmogorov-Smirnov test). (**B**) Receiver operating characteristic (ROC) curves, AUCs, and bootstrapped AUC 95% confidence intervals for final logistic regression model predictions on the discovery (blue) and validation (black) cohorts.

Because ensemble models offered only modestly higher training AUCs, we chose to use more-interpretable ridge-regularized logistic regression (Fig. S16), yielding a model defined by 6 logratios between five metabolites (labeled by *m/z* and retention time as 100.0@1.60, 126.0@5.04, 177.1@10.56, 302.0@15.94, and 318.9@20.08). Algebraic simplification from 6 logratios to the five underlying (log-transformed) metabolites yields regression coefficients for our final scoring function (Table 2). Model predictions are shown in Fig. 2A. The final model yielded an AUC of 89.4% (95% CI: 82.3–95.1) on the discovery cohort. The logratio derivation ensures the signature is inherently independent of absolute quantities and remains useful if sample dilution and/or instrument responses differ from those of the present study, provided the relative quantities of these five metabolites are accurately obtained.

**TABLE 2:** Logistic regression coefficients in the five-feature model

Molecular feature	Position	Coefficient*^a^*
126.0@5.04	Numerator	0.962
177.1@10.56	Numerator	0.793
318.9@20.08	Numerator	0.650
100.0@1.60	Denominator	−1.006
302.0@15.94	Denominator	−1.400

^*a*^ Coefficients do not sum to zero due to rounding.

### Signature validation

**Fig 3:**
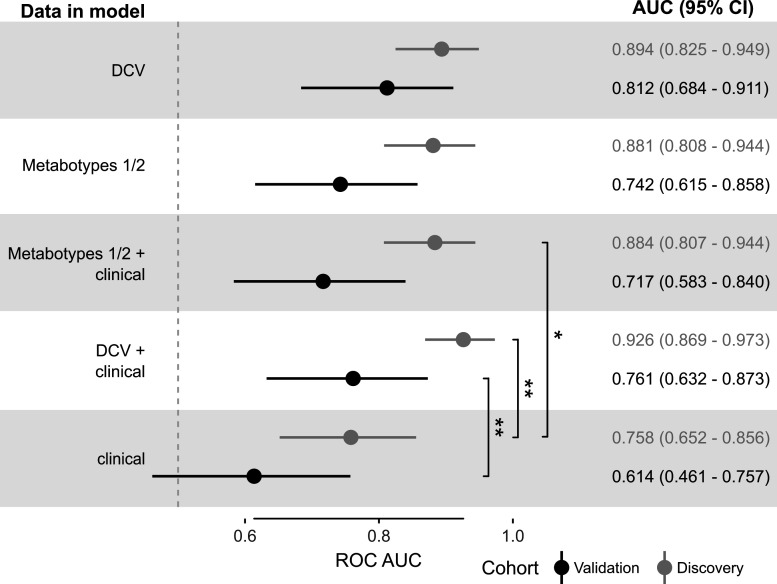
Performance of the final DiCoVar model (DCV) in the discovery and validation cohorts compared with models including clinical characteristics and with models excluding metabotype 3 (metabotypes 1/2). ROC AUC comparisons and 95% confidence intervals were computed by bootstrapping and corrected for multiple comparisons using Bonferroni’s method (20 comparisons within cohort, 5 comparisons between cohorts). Only significant comparisons are displayed (*P < 0.05, **P < 0.01). Vertical dashed line indicates 50% ROC AUC.

**Fig 4:**
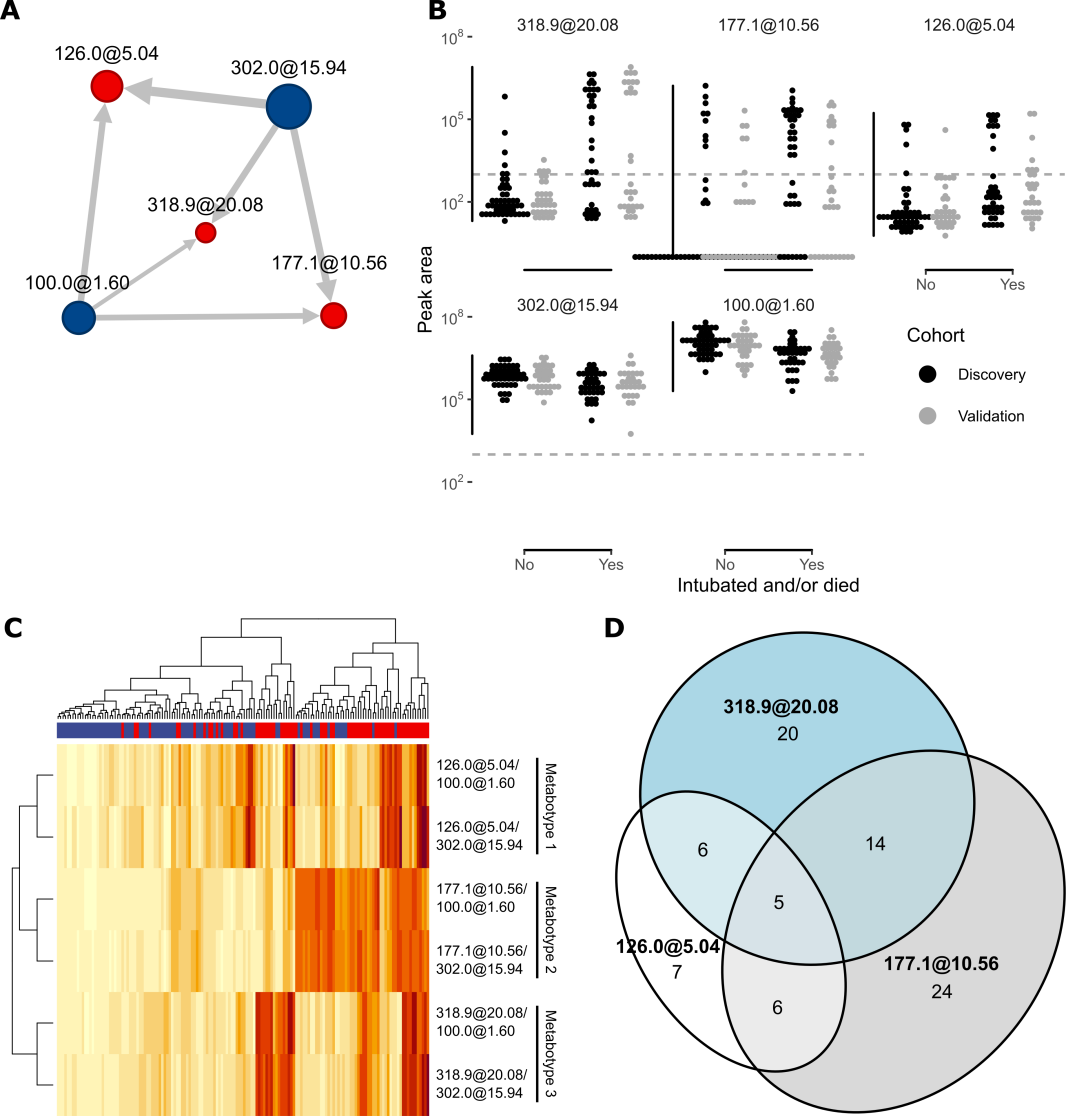
(**A**) Network representation of logratios in the final five-feature logistic regression model. Edges point from denominator to numerator. Edge thickness and vertex area represent absolute logistic regression coefficients for logratios and individual features, respectively. Red and blue vertices are associated with severe (positive coefficients) and non-severe (negative coefficients) outcomes, respectively. (**B**) Peak areas of biomarker metabolites in discovery and validation cohort samples as measured by LC-MS/MS. Dashed line indicates mass spectrometer limit of detection at 1,000 counts. (**C**) Heatmap of prognostic logratios for discovery and validation cohort samples, with row and column dendrograms based on hierarchical clustering with Euclidean distance and complete (maximum) linkage. Color scales are normalized for each row. Red and blue column headers indicate patients with severe and non-severe outcomes, respectively. (**D**) Euler diagram illustrating the degree of overlap between patient metabotypes. Membership was defined by the presence of each numerator metabolite over the 1,000-count limit of detection.

To evaluate its prospective external validity, we applied our severe COVID-19 signature model to the validation cohort. Instrumental analyses and data interpretation were conducted by operators blinded to the outcomes associated with each specimen. Upon unblinding, the validation cohort AUC for the severe COVID-19 signature (81.2%; 95% CI: 68.4–91.1) was significantly better than the null hypothesis (50%) (Fig. 2B) and was not significantly lower than in the discovery cohort (*P* > 0.05, bootstrap test; Fig. 3) (14). We observed similar distributions (*P* > 0.05, Kolmogorov-Smirnov test) of outcome classification probabilities in discovery and validation cohorts (Fig. 2A). Univariate analysis of individual logratios also revealed similar trends between discovery and validation cohorts (Fig. 4B; Fig. S19). Using a standard 50% probability cutoff to assign regression classifications yielded similar accuracy on the discovery and validation cohorts (81.6% vs. 74.6%; Table S3). Together, these results indicate minimal model overfitting and strong beyond-sample generalizability of the severe COVID-19 metabolomic signature.

### Three metabotypes define the metabolomic signature

A network representation (Fig. 4A) of the logratios between the five validated features distinguishes their roles as numerators (3 features) and denominators (2 features) in the model. The two denominator features were present in all specimens and are negatively associated with severe COVID-19 (Wilcoxon test, *P* < 0.01; Fig. 4B ), although the relative magnitudes of these associations are small. The denominators thus effectively serve as “housekeeping” features, providing ubiquitous physiological references for metabolite normalization, analogous to creatinine. In contrast, each of the three numerator features exhibits bimodal distributions, consistent with three distinctive severity-associated metabotypes (Fig. 4B) (15). Metabotype 1 is defined by the presence of 126.0@5.04, metabotype 2 by 177.1@10.56, and metabotype 3 by 318.9@20.08. Consistent with the manner of their selection, these metabotypes were not completely disjointed—subjects may simultaneously test positive for one or more metabotypes (Fig. 4C and D). Interestingly, metabotype 2 was associated with more advanced age (Wilcoxon test, *P* < 0.01; Fig. S20). No metabotype was associated with known clinical risk factors of diabetes or kidney disease (*P* > 0.05). These results are consistent with three independent biomarkers of COVID-19 severity with distinctive biochemical origins.

### Temporal relationships between metabotypes and severe COVID-19

**Fig 5:**
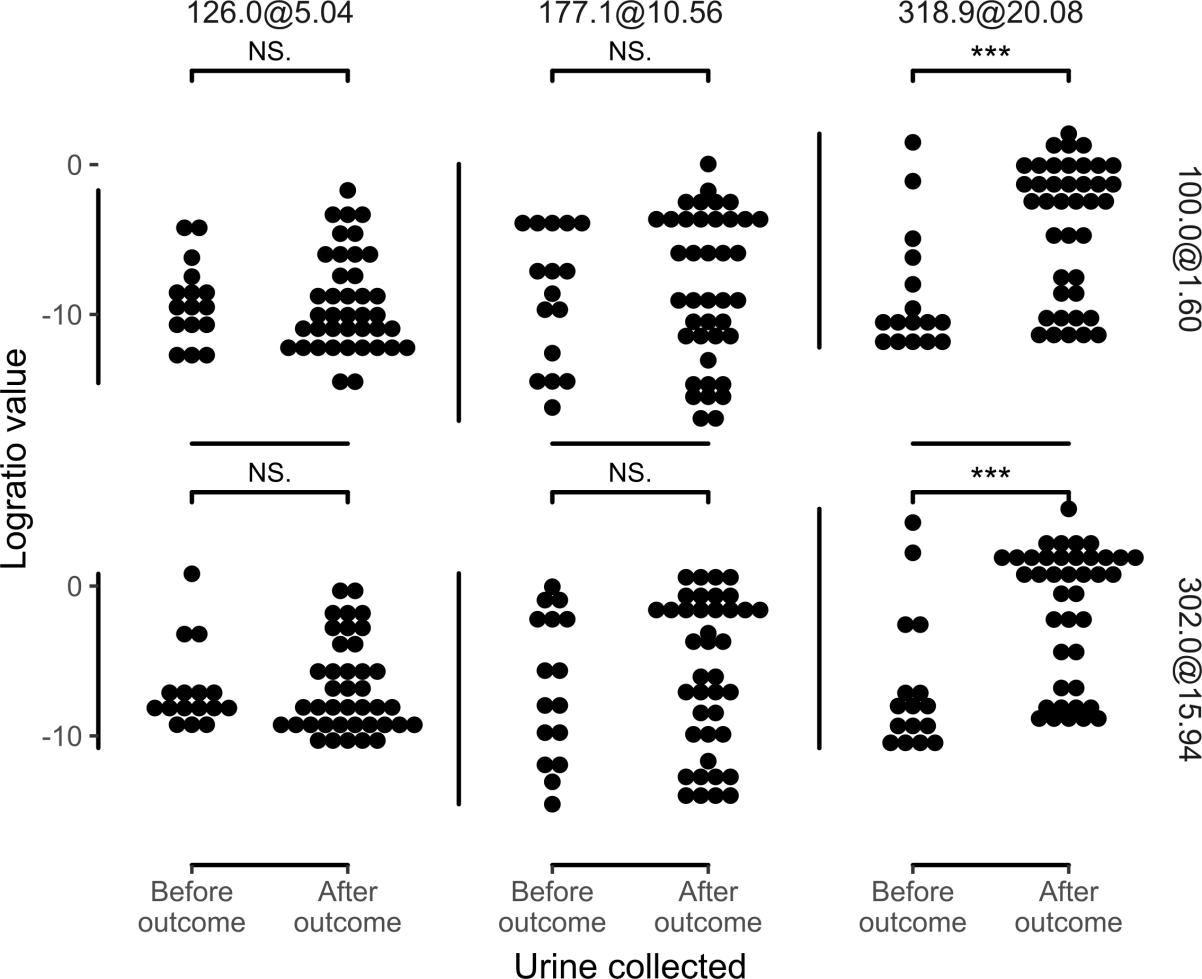
Model logratios with numerators 126.0@5.04 and 177.1@10.56 are not associated with urine collection date relative to outcome date, but numerator 318.9@20.08 is significantly elevated in patients with urines collected after outcome (Wilcoxon rank-sum test; ***P < 0.001, not significant [NS.] *P* > 0.05).

The potential for a metabotype to guide early antiviral interventions and patient disposition is influenced by its temporal relationship to severe disease, but this has not been typically considered in COVID-19 biomarker studies. Among the three metabotypes, the metabotype 3 signal was greater in specimens collected after intubation (Wilcoxon test, *P* < 0.01; Fig. 5). Metabotype 3 was neither uniformly absent in pre-intubation subjects nor uniformly present in post-intubation subjects, consistent with a physiological process that is closely related to acute respiratory failure. The presence of metabotypes 1 and 2, in contrast, was independent of intubation, consistent with baseline correlates of COVID-19 severity.

### Prognostic potential of metabotypes

COVID-19-associated metabotypes present early in infection, before the full inflammatory phase of illness, could potentially affect decisions about patient disposition and antiviral treatment. To evaluate their potential in this role, we fit predictor models including metabotypes 1 and 2 with or without baseline clinical predictor variables (diabetes, kidney disease, and age). The model using only metabotypes 1 and 2 was not significantly inferior to the model using all three metabotypes (Fig. 3, validation AUC 74.2% vs 81.2%; *P* > 0.05, bootstrap test with Bonferroni correction), consistent with significant predictive power in the absence of metabotype 3 (14). Adding clinical predictors non-significantly lowered the validation AUC versus metabotypes 1 and 2 alone. Notably, the validation cohort performance of clinical predictors alone was not significantly different from chance. These results suggest that metabotypes 1 and 2 are more consistently associated with severe COVID-19 than baseline clinical predictors.

### Chemical identification of housekeeper metabolites used as denominators

Additional information about COVID-19 progression may be obtained by chemically identifying the metabolites in the severe COVID-19 signature. Multistage fragmentation (MS^n^) coupled with high-resolution accurate mass analysis of the five metabolites comprising our signature revealed they are oxyhydrocarbons, three of which possess a single nitrogen and no other heteroatoms. The two denominator metabolites in our model were identified with high confidence as an O-acylcarnitine (302.0@15.94) and δ-valerolactam (100.0@1.60) (Table 3). The fragmentation pattern of feature 302.0@15.94 contains multiple diagnostic fragments observed in other acylcarnitines (Fig. S2 to S5), with differences attributable to the presence of an unsaturated nine-carbon fatty acyl group. Feature 100.0@1.60 was definitively confirmed by comparison to authentic δ-valerolactam, an established urinary metabolite generated from cadaverine and subject to cytochrome P450-dependent metabolism (Fig. S6) (16).

**TABLE 3:** Final prognostic model features and putative identification confidence

Feature	Confidence	Putative identity	ESI + mass	RT (min)	Neutral composition
302.0@15.94	Confident class ID	O-Nonanoylcarnitine	302.2327	15.94	C_16_H_31_NO_4_
126.0@5.04	Confident ID	Levetiracetam metabolite	126.0906	5.04	C_7_H_11_NO
177.1@10.56	Approximate chemical class	Similar to 1-phenyl-2-hexanone	177.12704	10.56	C_12_H_16_O
318.9@20.08	Approximate chemical class	Similar to hydroxyenterodiol	319.152	20.1	C_18_H_22_O_5_
100.0@1.60	Confirmed with reference std.	Delta-valerolactam	100.0757	1.6	C_5_H_9_NO

### Metabotype 1 metabolite identification

The metabolite-defining metabotype 1, 126.0@5.04, was identified with high confidence as an electrospray ionization source-decay fragment of a previously-observed deaminated metabolite of the anticonvulsant drug levetiracetam (Keppra) (Fig. S7 to S10) (17). Its MS2 spectrum exactly matched a reference spectrum of levetiracetam in the mzCloud spectral library, while its molecular weight matches the deaminated metabolite. Concomitant detection of unmetabolized levetiracetam in specimens with this feature further increased confidence in this assignment (Fig. S10). This result is consistent with a positive association between levetiracetam use and progression to severe COVID-19.

### Metabotype 2 metabolite identification

The metabotype 2 feature, 177.1@10.56, has predicted molecular formula C_12_H_16_O (Table 3) and shares multiple MS2 fragments with the fecal metabolite 1-phenyl-2-hexanone (Fig. S11A), including a tropylium ion characteristic of carbon-substituted benzene rings. Moreover, the 131.0850 *m/z* MS2 fragment ion has a neutral loss of C_6_H_6_ (consistent with benzene) in its MS3 spectrum (Fig. S11B). An H_2_O neutral loss in the MS2 spectrum also suggests the presence of an alcohol. Together, these data are consistent with 177.1@10.56 as a monounsaturated aliphatic alcohol-substituted benzene. The specific biochemical origin of this metabolite is unclear, though the presence of a benzene ring may implicate phenylalanine or tyrosine metabolism.

### Metabotype 3 metabolite identification

The metabotype 3 feature, 318.9@20.08, with predicted molecular formula C_18_H_22_O_5_, yielded no direct match in online spectral databases. However, it shares distinctive fragments and neutral losses with enterodiol (Fig. S12 to S14), the eponymous member of an established group of human urinary metabolites and a product of lignan metabolism by the intestinal microbiota. The predicted formula (Table 3) matches hydroxyenterodiol, which may derive from P450-mediated hydroxylation of enterodiol in mammalian hosts (18, 19). These data are consistent with 318.9@20.08 as a member of the enterodiol family of bacterial-human co-metabolites derived from common dietary compounds. Elevation of this metabolite after respiratory failure in a subset of patients with severe COVID-19 may reflect increased systemic absorption associated with increased gut barrier permeability and/or increased human P450 metabolism of xenometabolites.

## DISCUSSION

In this study, we describe an accessible, patient-based approach to rapid prognostic LC-MS/MS assay development for a new pandemic infection. If completed in time for use with COVID-19 patients in 2020, this approach might have improved triage decisions and optimized allocation of limited convalescent plasma and remdesivir. Entities charged with pandemic preparedness should consider expanding diagnostic efforts beyond pathogen identification alone to include prognostic assays compatible with existing laboratory equipment.

Relative to previously published metabolomic studies in COVID-19 patients, this study is distinguished by its clinically valid cohorts of patients presenting for medical evaluation, use of instrumentation accessible to clinical laboratories, feature selection yielding a simplified metabolite signature, blinded prospective validation, and careful attention to the relationship between metabolites and endpoint timing (20–23). In the context of a new pandemic, accessibility of specimens prior to severe symptoms is challenging. With limited resources, we elected to implement this study in a hospital setting where tests were available, specimens could be safely collected, and outcomes could be reliably discerned. Because we reasoned that stable predictive markers would persist in patients after intubation, we elected to include subjects with post-intubation specimens in our cohorts. Inclusion of these specimens ensured timely recruitment of subjects and permitted temporal analyses (Fig. 5) to identify lagging, non-predictive, indicators such as metabotype 3. When the 20 post-intubation specimens (32% of subjects) were excluded from the validation cohort, the model exhibited a non-significant trend (receiver-operator characteristic area under the curve [ROC AUC] 68.2%, 95% CI: 49.4–84.6) toward prediction of severe outcomes. Given that metabotypes 1 and 2 are not temporally associated with intubation (Fig. 5), we regard this result as a probable Type II error. We advise future biomarker studies to carefully monitor the timing of specimen collection relative to key events in infection pathophysiology. Future studies may also consider longer term follow-up to identify markers for post-infectious sequelae. Any of the three severity-associated metabotypes identified here could conceivably modify the risk of post-COVID conditions, which are affected by the course of early infection (24–27).

Several aspects of our approach are critical to its utility in a future pandemic. Primary among these is prompt establishment of an approved protocol for collecting specimens and longitudinal clinical characteristics. In the present study, this began in early March 2020, anticipating a surge of cases and a limited supply of antiviral therapies. The delay of the present study resulted in part from the recent development of the data analysis methods used here (12, 13, 28) and the need to curate the patient data, though other methods could have potentially been considered (11, 29). The present report was also delayed by efforts to determine the identities of metabolites used in the prototype assay, which we felt would be useful for interpreting the results. We calculate that our present approach would require 100–200 patients in a similar future pandemic, assuming that prognostic metabolites exist and exhibit log-normal distributions (Fig. S24). More advanced mass spectrometers with reduced data variability could reduce study size requirements. Specimen accrual and disease-specific patient data collection are likely to be rate-limiting steps.

Each of the three severe COVID-19 metabotypes suggests unique insights into physiologic processes or exposures that affect COVID-19 severity. The levetiracetam metabolite defining metabotype 1 was unexpected and raises important questions about its association with COVID-19 severity. Of the two subjects newly treated with levetiracetam prior to sample collection, only one had generalized seizure attributed to COVID-19. The majority (22/24, 92%) of subjects with detectable levetiracetam metabolite had been receiving it for seizure prophylaxis. Levetiracetam may be a marker for patient conditions that predispose to COVID-19 progression, a direct biological modifier of COVID-19 progression risk, or a combination thereof. Indeed, early levetiracetam efficacy trials conducted before the COVID-19 pandemic identified a positive association between levetiracetam administration and other respiratory infections (30). The levetiracetam metabolite does not exclusively drive the present model, which remains accurate even after holding out patients in whom it was detected (Fig. S21). Further study, such as a retrospective patient data analysis, would be necessary to determine the degree to which seizure disorders or related conditions, levetiracetam, or a class effect among anticonvulsants contribute to COVID-19 severity risk.

The nature of metabotype 2 remains unclear. While previously detected in human feces (31), 1-phenyl-2-hexanone is not clearly an intestinal or microbiome-derived product. Notably, metabotype 2 exhibits a distinctive association with age, a frequently applied proxy for COVID-19 risk. Its trend toward superiority over age as a severity predictor (Fig. S25) raises the possibility that it represents an age-associated physiological process that modifies COVID-19 risk. Given the widely noted role of age as a risk factor in the COVID-19 pandemic, the nature of this metabotype merits additional study.

Because metabotype 3 was preferentially detected in patients with established respiratory failure, we did not regard it as a strong prognostic candidate. This affects the metabotype’s potential to inform COVID-19 antiviral therapy, which is effective in most patients only when administered early in disease, as with other acute respiratory viruses (4, 6, 32, 33). Hydroxyenterodiol, the tentatively-identified feature defining metabotype 3, is derived from enterodiol, which is produced by the intestinal microbiota. It is plausible that intestinal inflammation in some forms of severe COVID-19 leads to increased enterodiol permeability and increased hydroxylation by host P450 enzymes. If metabotype 3 represents an inflammatory endotype in COVID-19 patients, it may instead help inform selection of immune modulating therapies (34, 35).

Limitations of this single-site study include its generalizability to other sites and its prospective applicability, both likely concerns for any evolving pandemic illness. At the time of this writing, population-wide vaccine and convalescent immunity to SARS-CoV-2 and antiviral supply is more robust than that in 2020, limiting the current clinical value of the prototype assay. This could change rapidly if a new SARS-CoV-2 variant emerges that evades current immunity or antiviral agents. Continued virus evolution and improved treatment strategies may also lessen the prospective validity of an assay based on results from a single site early in the pandemic. An intrinsic limitation of metabolomic data analysis is that choices made during machine learning may influence the set of biomarkers discovered, especially when multiple near-optimal sets exist. Preliminary tests (Fig. S22 and S23) suggest that the biomarker signature presented here is relatively robust to these choices. More sophisticated but less widely available instrumentation may have identified different or more complex signatures with superior sensitivity or specificity but more limited applicability. In a future pandemic, some of these limitations may be remedied by coordination between sites and access to improved instrumentation.

## MATERIALS AND METHODS

### Patient population

Patients presenting to the Barnes-Jewish Hospital emergency department with symptoms compatible with COVID-19 or at high risk of developing COVID-19 were enrolled upon submission of a nasopharyngeal swab for SARS-CoV-2 RNA testing using a Food and Drug Administration-approved clinical PCR test (Fig. S1). The primary endpoint for progression of COVID-19 to severe disease was respiratory failure requiring mechanical ventilation and/or death within 90 days of study enrollment. Subjects enrolled from 1 March 2020 to 30 June 2020 comprised the initial discovery data set (*n* = 96, SARS-CoV-2 positive *n* = 87). Additional subjects enrolled between 1 July 2020 and 30 September 2020 comprised the validation data set (*n* = 67, SARS-CoV-2 positive *n* = 63) (Table 1). The workflow for discovery and validation cohorts is outlined in Fig. S1. Importantly, no information about the validation cohort was used in identifying and formulating our candidate metabolomic signature. Clinical data were extracted from electronic health records by the Washington University Institute of Informatics and provided in a de-identified manner.

### Urine collection and processing

Patient urine samples were collected in a sterile cup by the hospital staff as convenient and transported twice a day to the laboratory. Samples were stored at 4°C until ready for processing on the same day. All samples were processed and stored in less than 24 hours. Urine was filtered through a 40-µm mesh followed by 2,000 × *g* centrifugation for 12 minutes. Supernatant was immediately aliquoted into cryovials and stored at −80°C until ready for experimental analysis.

### Liquid chromatography-mass spectrometry

Urinary metabolites were measured using an AB Sciex 4000 QTrap triple-quadrupole mass spectrometer interfaced with a Shimadzu HPLC as previously described (36, 37), (see supplemental material for details). Reversed-phase chromatography was conducted with an Ascentis-Express fused core phenyl-hexyl column (Millipore Sigma; 100 mm × 2 mm × 2.7 µm). Metabolite profiling was conducted in full-scan mode across a mass/charge range of 50–1,200 *m/z*. Multiplexed profiling of selected metabolites was conducted in MS/MS mode. Structural characterization of validated metabolites was performed using an Orbitrap ID-X high-resolution accurate mass spectrometer coupled to a Vanquish ultra-high pressure liquid chromatograph (UHPLC; Thermo Scientific), as described in the supplemental materials (Fig. S2 to S14).

### Metabolomic feature filtering and expression as logratios

To focus our search on broadly applicable features, we restricted analyses to the 103 negative-ion and 163 positive-ion features present in >50% of samples in at least one clinical outcome group. To control for physiologic differences in urinary dilution, we analyzed urinary metabolomic profiles as log-transformed metabolite peak area ratios (hereafter designated “logratios”) and selected from all possible ratios in the data during subsequent feature selection steps (11, 29). Using metabolite ratios allowed us to identify urinary reference metabolites without making *a priori* assumptions, for example, we did not assume that any single metabolite, such as creatinine, would provide an optimal physiologic reference.

### Bounded feature selection and machine learning modeling

To select a reduced set of LC-MS metabolite features for targeted LC-MS/MS analysis, we used a feature selection approach based in genetic algorithms (for additional details, see supplemental materials). To estimate the ability of metabolomic and/or clinical data to correctly classify severe outcomes and to minimize overfitting, we performed this process with fivefold cross-validation train/test splits of the discovery cohort, repeated 20 times with different random seeds, for each combination of metabolomic data and clinical metadata. After a subset of metabolites was identified using the genetic algorithm approach, we used differential compositional variation scores to select subsets of logratios for model training through multistage feature selection using network-based, recursive elimination combined with penalized regression techniques (28). This broad approach was used to improve model performance and improve interpretability by eliminating uninformative logratios. Clinical metadata features were optionally selected using the Boruta algorithm on individual training partitions (38). Features selected in 8 of 10 repeats of the Boruta algorithm were retained. The resulting clinical metadata subset was then appended to the selected metabolite subset. Finally, the XGBoost algorithm was used to train tree booster models using the selected features (39).

Across all test splits, average model AUC was superior for models combining metabolite and clinical data. Models using positive-mode data, negative-mode data, or both modes yielded similar average model AUCs, with larger models generally exhibiting higher AUCs (Fig. S15). Because positive mode ionization is more common and sensitive, we proceeded with a model combining positive mode features and clinical metadata. To select a smaller set of representative metabolites and clinical attributes, we repeated the feature selection process 50 times with LC-MS features and clinical attributes, with a maximum model size of 70 variables. We aggregated the importance scores for each selected variable across all models and, representing each subset as a binary vector, selected the subset with minimum total pairwise Hamming distances as the most representative (Fig. S22). The 24 metabolites in this most representative subset were selected for targeted metabolomic analysis.

### Metabolite signature selection and final prognostic model

To simplify the 24-metabolite signature while maintaining classification performance, we used the targeted differential compositional variation machine learning framework implemented in the DiCoVarML R package (13). DiCoVarML is a multilevel nested framework that respects the variable dilution of urinary specimens by leveraging network methods that identify and robustly estimate classification performance of minimal logratio signatures (13). Pairwise logratios between the 24 metabolites were calculated with zeroes imputed using multiplicative replacement (28, 40). We selected ridge regression over ensemble models for easier interpretability and because we did not see substantial decrease in accuracy (Fig. S16). As in the previous stages of feature selection and model building, only discovery cohort data were used as input. Out-of-sample classification performance was estimated using five repeats of twofold cross-validation for each target subset size (four to eight metabolites). Multiplicative zero imputation was performed separately for each cross-validation step to avoid leaking information between folds. The DiCoVarML nested classification performance estimate was then used to select the final subset of five metabolites (Fig. S16) and train the final ridge regression model with the corresponding pairwise logratios. For ease of interpretation, the model in terms of 6 logratios was algebraically simplified to 5 log-scaled metabolite signal values to specify our final prognostic function (coefficients in Table 2). This final prognostic function was applied to LC-MS/MS data to predict outcome severity in the validation cohort.

### Statistics

All statistical analysis and machine learning model building and assessment were done in R version 4.1.0. The R mixOmics package was used for PCA (41). We used the R glmnet package to predict outcome probabilities and labels using the final model trained on the discovery data (42). Discovery and validation set AUCs and 95% confidence intervals were computed using the R pROC package (14). ROC AUCs were compared using bootstrapping (R pROC package, roc.test function). Comparison *P* values were adjusted for multiple comparisons by the Bonferroni method (R stats package, p.adjust function). The prognostic function scores, using the discovery-trained model, were computed for both validation and discovery data using the R predict function with response output. The Kolmogorov-Smirnov test (R stats package, function ks.test) was used to compare distributions of predicted probabilities of severe outcome between discovery and validation cohorts, stratified by outcome. The two-sample Wilcoxon signed-rank test (R stats package, function wilcox.test) was used to describe differences in means of logratio features between outcome groups.

## Data Availability

De-identified clinical metadata, processed metabolomic data, and associated data analysis scripts may be obtained from the corresponding author upon request.

## SUPPLEMENTAL MATERIAL

Supplemental Material mbio.03343-23-s0001.docx Supplemental methods, Fig. S1 to S25, and Tables S1 to S3.

ASM does not own the copyrights to Supplemental Material that may be linked to, or accessed through, an article. The authors have granted ASM a non-exclusive, world-wide license to publish the Supplemental Material files. Please contact the corresponding author directly for reuse.
