# Supplementary material for: Development of a metabolome-based respiratory infection prognostic during COVID-19 arrival
Source: mBio. 2024 Nov 22;16(1):e03343-23. doi: 10.1128/mbio.03343-23 (PMC11708037; doi:10.1128/mbio.03343-23)
Supplement: Supplemental Material — Supplemental methods, Fig. S1 to S25, and Tables S1 to S3. [file mbio.03343-23-s0001.docx]

**Figure S1.** Cohort assembly and data analysis workflow.

# Supplemental LC-MS methods

Samples were injected (10 μL) using a Shimadzu UHPLC and separated on an Ascentis-Express fused core phenyl-hexyl column (Millipore Sigma; 100 mm x 2 mm x 2.7 μm) using a flow rate of 0.35 mL/min. The column was maintained at 45°C. Proportions of mobile phase solvents A (0.1% formic acid in H2O) and B (90% acetonitrile, 0.1% formic acid in H2O) were varied according to the gradient in **Table S1A**. The column was equilibrated at 2% solvent B for 3 minutes between samples. Patient urine metabolites were measured using an AB Sciex 4000 QTrap triple-quadrupole mass spectrometer. Samples were analyzed in positive- and negative-ion modes using a Turbo V electrospray ionization source with ion spray voltage set to 4500 V and heater temperature at 600°C. Ion source gas 1 and 2 were set to 45 and curtain gas set to 35. Collision gas flow was set to “low”. Molecular ions were monitored in enhanced MS1 (EMS) mode with declustering potential (DP) of +/-60 V across a mass/charge range of 50-1200 m/z. To compare metabolomes between specimens, LC-MS profiles from each specimen in the discovery cohort were aligned using MarkerView version 1.2.0 (Sciex). This generated a set of metabolite features defined by a characteristic mass/charge ratio and LC retention time.

The 24-metabolite putative biomarker panel (see description of bounded feature selection in the main text) was subsequently measured by liquid chromatography-multiple reaction monitoring (LC-MRM) using the precursor/product-ion pairs listed in **Table S2**. Injection volume was set to 5 μL. The HPLC gradient timing was optimized to more quickly and effectively separate these targeted metabolite features (**Table S1B**). Total flow rate was increased to 0.45 mL/min and column temperature was increased to 55°C. All other HPLC and ion source parameters were kept the same. For all metabolites, EP and CXP were set to 10 and 12 V, respectively. Peak areas were integrated using AB Sciex Analyst software version 1.6.3.

Biomarker metabolites were characterized using an Orbitrap ID-X high-resolution accurate mass spectrometer coupled to a Vanquish UHPLC (Thermo Scientific). HPLC parameters were identical to those used for LC-MRM analysis on the QTrap instrument. Metabolites of interest were ionized by an H-ESI ion source with spray voltage set to +3400 V, sheath gas set to 50, auxiliary gas set to 10, and sweep gas set to 1. The ion transfer tube was held at 325°C and the vaporizer at 350°C. Standard MS2/MS3 method templates were optimized as needed for particular metabolites. Data was collected using Xcalibur software and aligned using Compound Discoverer 3.1 (Thermo Scientific). Detailed interrogation of spectral data and prediction of molecular formulas was performed using FreeStyle and MassFrontier software (Thermo Scientific), as described in the following section of the supplemental materials (**Figures S2-S14**). Metabolite spectra were queried against the mzCloud online spectral library to identify matches or similar compounds (Thermo Scientific).

**Table S1**

**A**. HPLC gradient separates prognostic biomarkers.

| Time (min) | % buffer B |
| --- | --- |
| 0 | 2% |
| 1 | 2% |
| 23 | 35% |
| 33 | 98% |
| 36 | 98% |
| 37 | 2% |
| 40 | 2% |

**B.** Optimized HPLC gradient for separating prognostic biomarkers.

| Time (min) | % buffer B |
| --- | --- |
| 0 | 2% |
| 1 | 2% |
| 12.5 | 19.3% |
| 23 | 30% |
| 25 | 98% |
| 28 | 98% |
| 29 | 2% |
| 30 | 2% |

**Table S2.** Candidate correlative biomarkers monitored by LC-MS/MS.

| Feature | RT (min) | Precursor (Da) | Product (Da) | DP (V) | CE (V) |
| --- | --- | --- | --- | --- | --- |
| 100.0@1.60 | 1.6 | 100 | 100 | 60 | 5 |
| 126.0@5.04 | 5.04 | 126.03 | 93 | 80 | 20 |
| 166.1@1.27 | 1.27 | 166.05 | 135 | 100 | 20 |
| 177.1@10.56 | 10.56 | 177.11 | 159 | 40 | 20 |
| 181.0@3.36 | 3.36 | 181 | 124 | 40 | 20 |
| 188.0@2.13 | 2.13 | 188.02 | 145.9 | 80 | 20 |
| 227.0@3.61 | 3.61 | 227 | 199.8 | 60 | 20 |
| 241.1@3.61 | 3.61 | 241.07 | 183 | 40 | 20 |
| 253.1@17.25 | 17.25 | 253.06 | 197 | 80 | 20 |
| 265.0@4.96 | 4.96 | 264.97 | 136 | 40 | 20 |
| 288.1@14.52 | 14.52 | 288.08 | 85 | 40 | 50 |
| 302.0@15.94 | 15.94 | 302.03 | 243.1 | 60 | 20 |
| 310.0@15.14 | 15.14 | 309.97 | 85 | 40 | 50 |
| 318.9@20.08 | 20.08 | 318.9 | 231 | 60 | 20 |
| 320.9@14.90 | 14.9 | 320.94 | 275 | 40 | 20 |
| 337.9@11.97 | 11.97 | 337.92 | 141 | 40 | 80 |
| 371.1@7.20 | 7.2 | 371.12 | 324.9 | 40 | 20 |
| 413.0@1.61 | 1.61 | 412.97 | 412.97 | 40 | 5 |
| 413.8@4.70 | 4.7 | 413.84 | 241 | 60 | 50 |
| 451.1@9.58 | 9.58 | 451.12 | 393 | 125 | 53 |
| 459.1@15.15 p325 | 15.15 | 459.13 | 325 | 80 | 50 |
| 459.1@15.15 p283 | 15.15 | 459.13 | 283 | 60 | 20 |
| 555.3@24.77 | 24.77 | 555.31 | 189.1 | 40 | 50 |
| 565.2@15.82 | 15.82 | 565.24 | 389.1 | 40 | 50 |

# Characterization of metabolites

## Feature 302.0@15.94

The MS2 spectrum of 302.0@15.94 contains many distinctive acyl-carnitine features **(Figure S2).** The 243 m/z product ion results from the neutral loss of trimethylamine from the carnitine moiety. Furthermore, the 144, 85, and 60 m/z product ions are all fragments of carnitine that appear in other acyl-carnitine reference standards **(Figure S3)**. Based on the predicted molecular formula (C_16_H_31_NO_4_), the acyl group contains 9 carbons and no unsaturation. Some patient samples contain multiple isomers of 302.0@15.94 with slightly different retention times (**Figure S4**). The MS3 fragmentation patterns of MS2 ion 243 m/z are different but related between isomers, suggesting that these are structural isomers of 302.0@15.94 (**Figure S5**). The presence of apparent structural isomers and absence of acyl group unsaturation implies that at least some of these isomers have branched acyl chains.


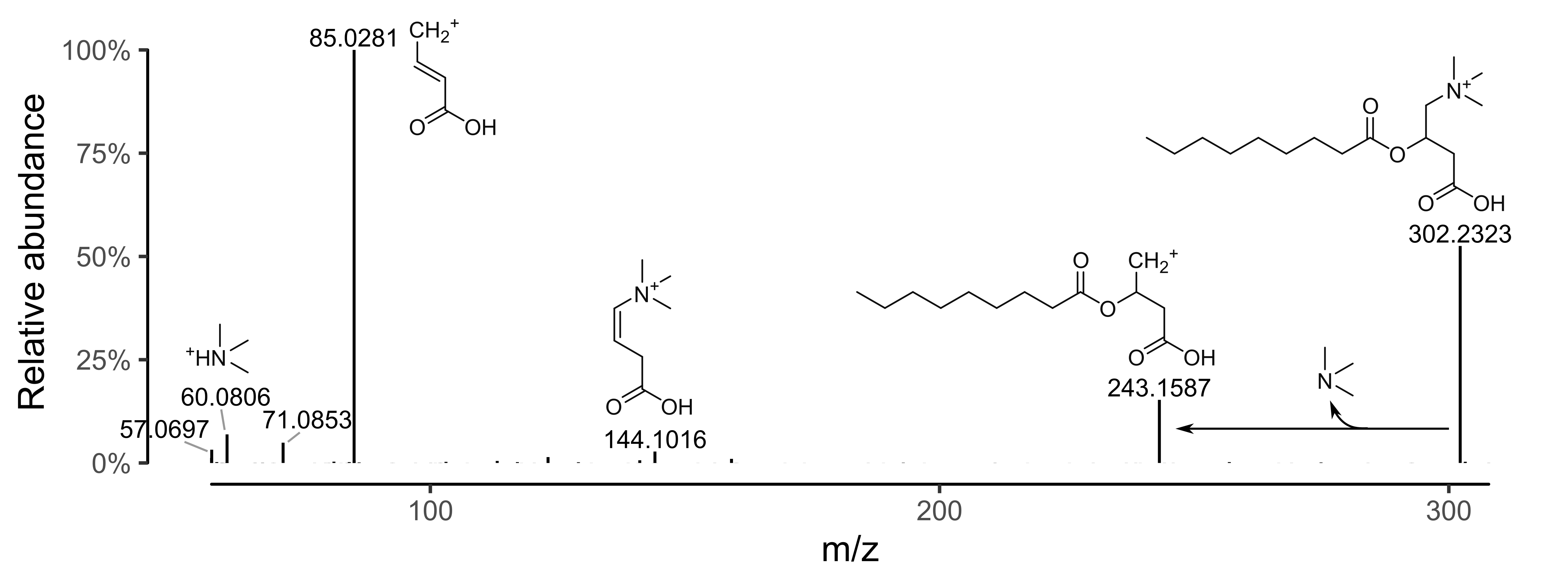


**Figure S2:** MS2 spectrum of feature 302.0@15.94. Predicted fragment structures are included for several distinctive carnitine features. The neutral loss from 302 m/z to 243 m/z corresponds to loss of trimethylamine.


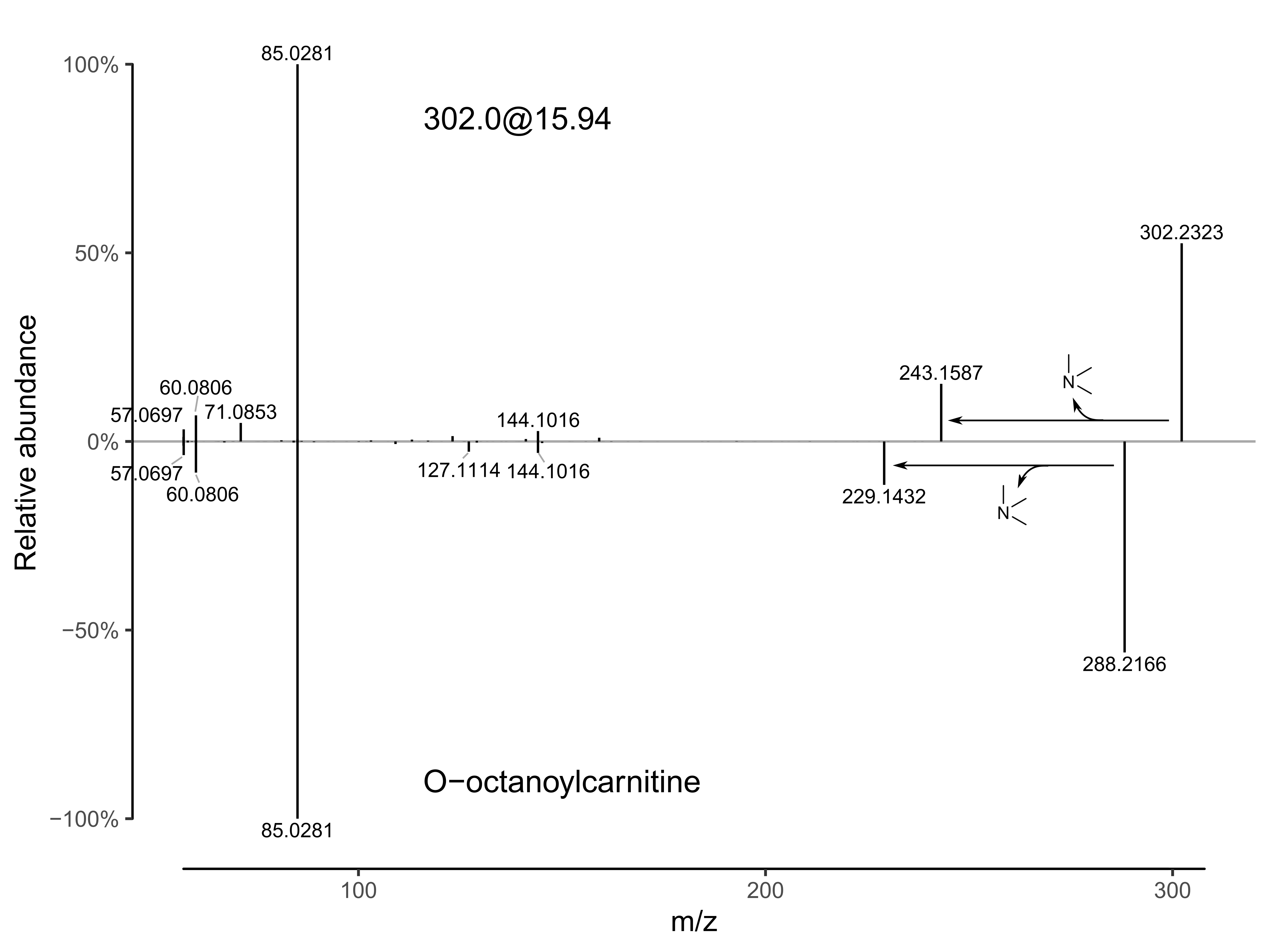


**Figure S3:** Comparison of MS2 spectra from 302.0@15.94 and an authentic standard of O-octanoylcarnitine. Note matching carnitine fragments (144, 85, and 60 m/z) and neutral loss of trimethylamine.


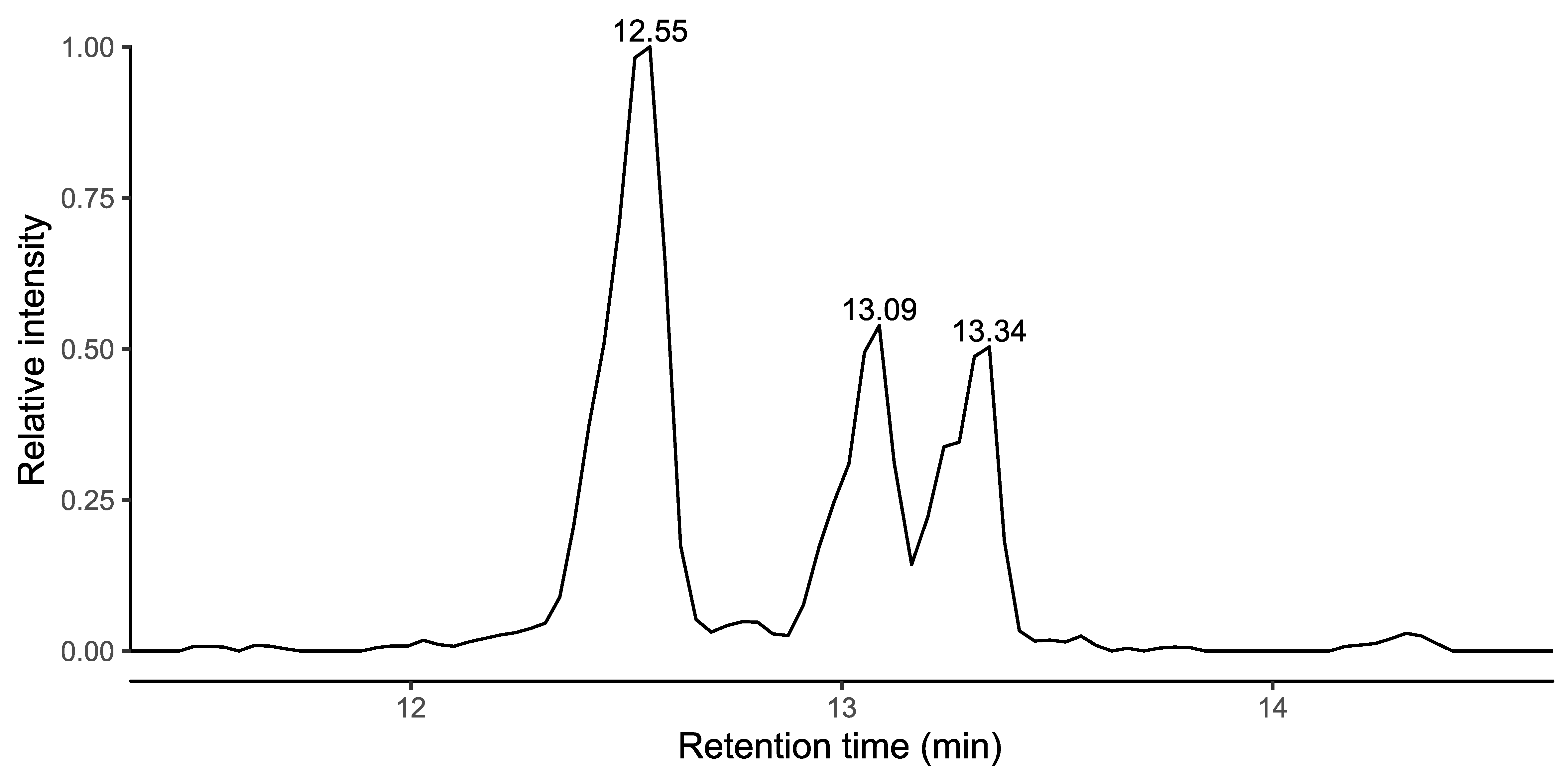


**Figure S4:** Extracted-ion chromatogram of MS2 fragment 243 m/z. Feature 302.0@15.94 elutes at 12.55 minutes and two isomers elute at 13.09 and 13.34 minutes.


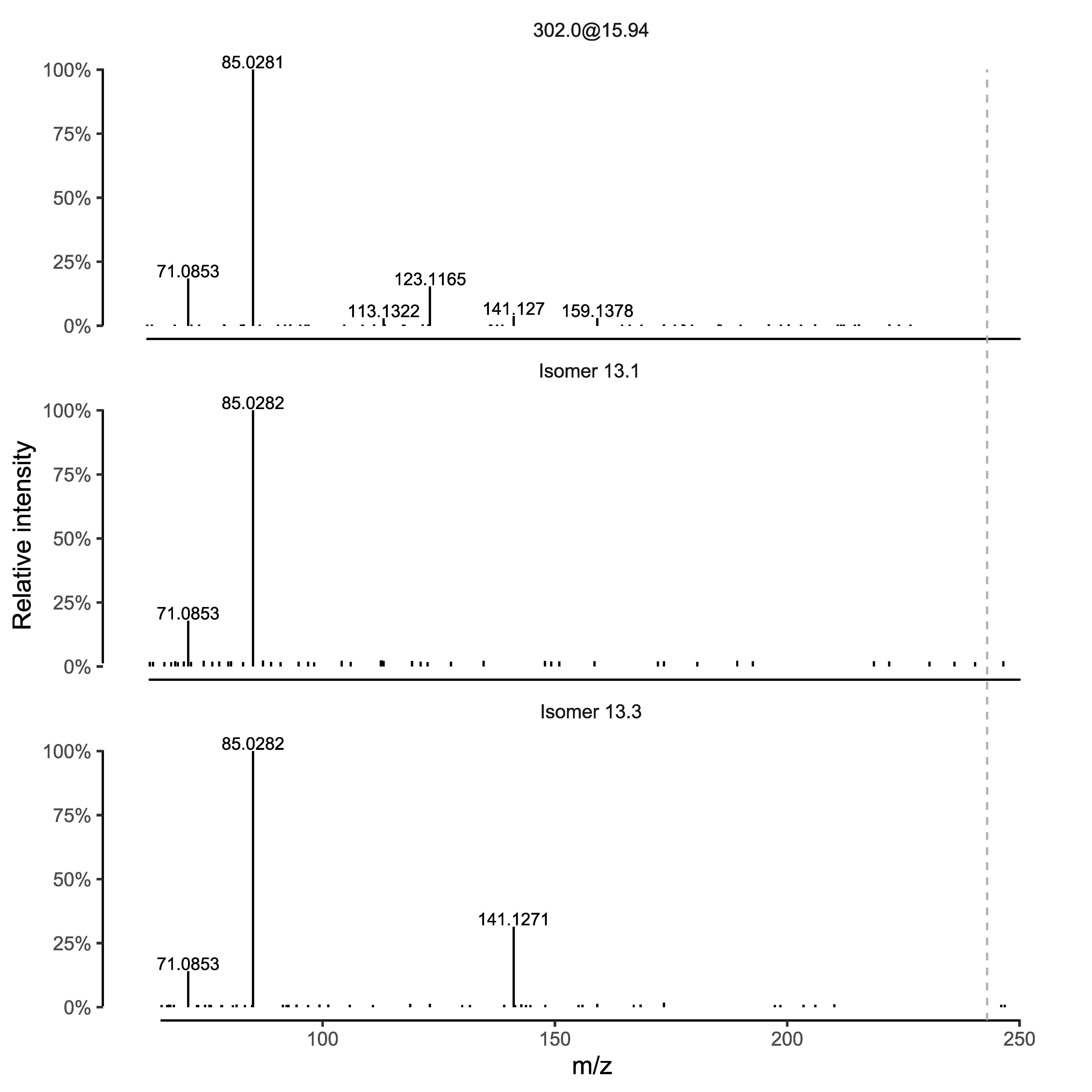


**Figure S5:** Comparison of MS3 spectra from MS2 fragment 243 m/z from the three isomers (**Figure S4**). Dashed vertical line indicates precursor ion m/z ratio.

## Feature 100.0@1.60

The MS2 spectrum of feature 100.0@1.60 exhibited strong similarity to an MS2 spectrum of delta-valerolactam in the mzCloud spectral library (Thermo Fisher). A reference standard of delta-valerolactam exhibited the same tandem MS/MS (MS^2^) spectrum and retention time as the 100.0@1.60 feature (**Figure S6A and B**), confirming its identity.

**A.**


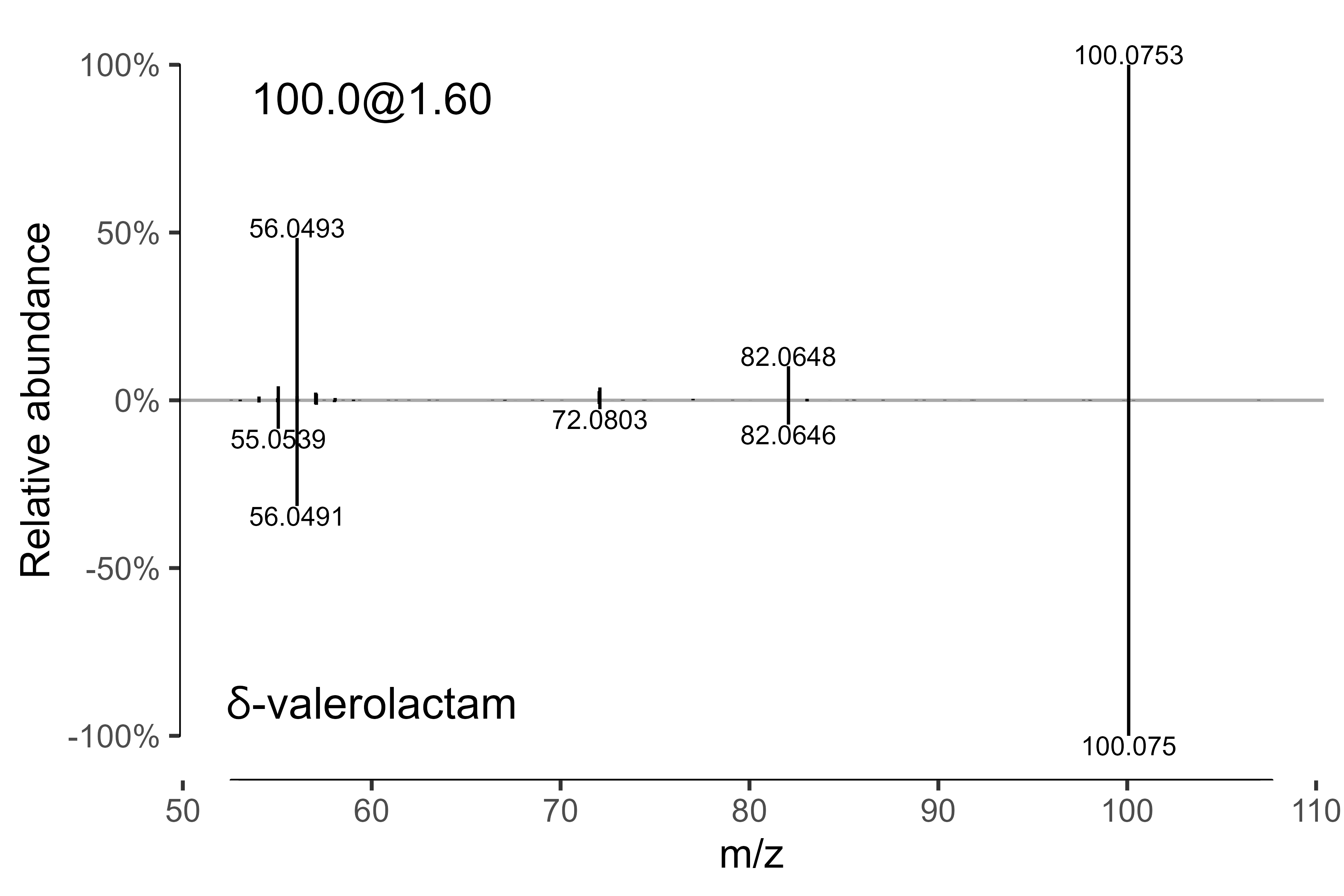


**B.**


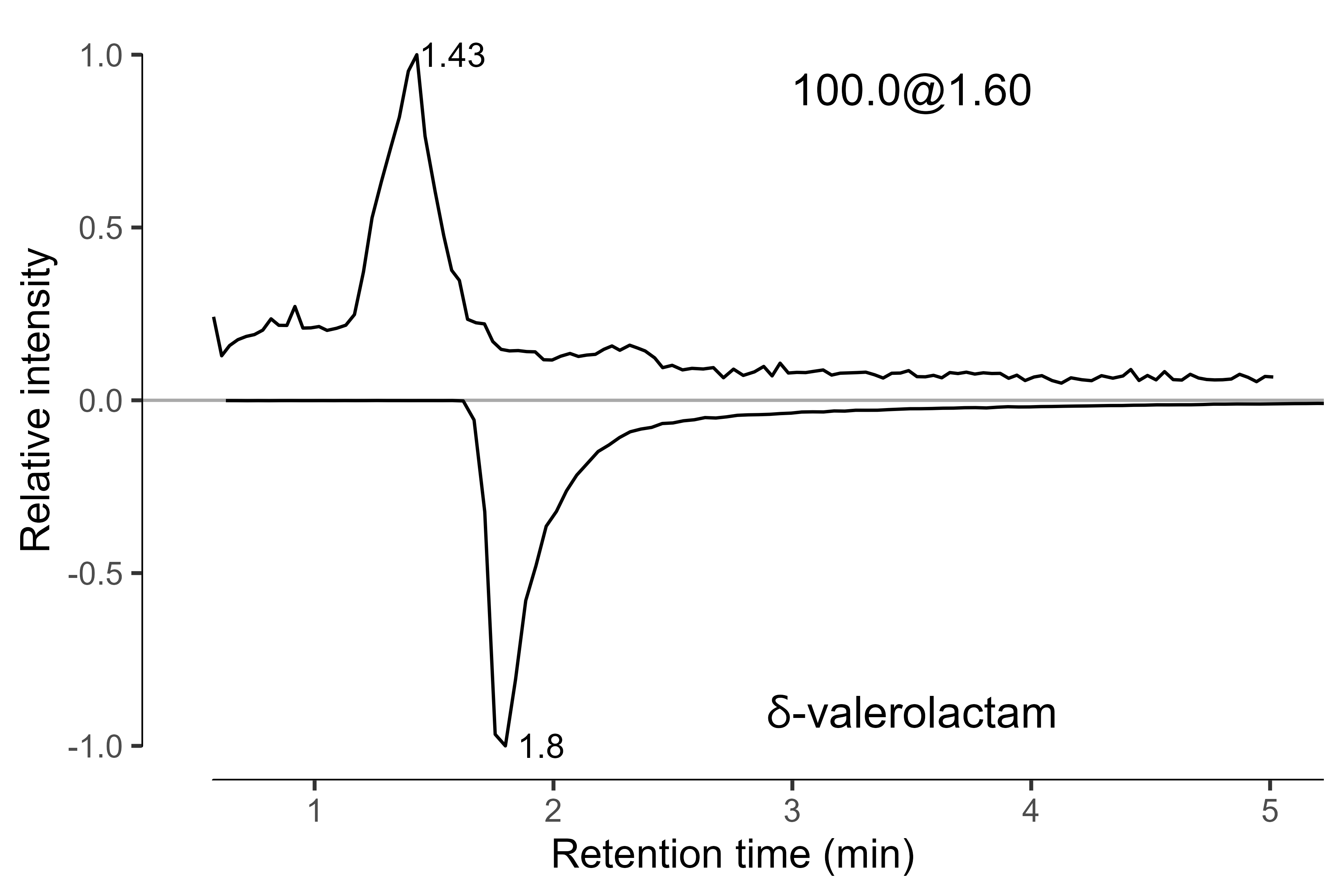


**Figure S6: A.** MS^2^ spectra of the 100.0@1.60 feature and delta-valerolactam. **B.** Extracted-ion chromatograms (XICs) of 56.049 m/z MS2 fragment showing elution times of 100.0@1.60 and delta-valerolactam. Chromatography conditions were as described in the main text.

## Feature 126.0@5.04

The MS2 spectrum of feature 126.0@5.04 matched the spectrum of levetiracetam in the mzCloud library **(Figure S7)**. However, the molecular ion (126.0910 m/z) of this feature was 45.0218 Da lower than for levetiracetam (171.1128 m/z). The mass and isotopologue patterns of 126.0@5.04 predict a molecular formula of C_7_H_11_NO, which is a subset of the formula of levetiracetam (C_8_H_14_N_2_O_2_). Taken together, this suggested that 126.0@5.04 is a source-decay fragment of levetiracetam.


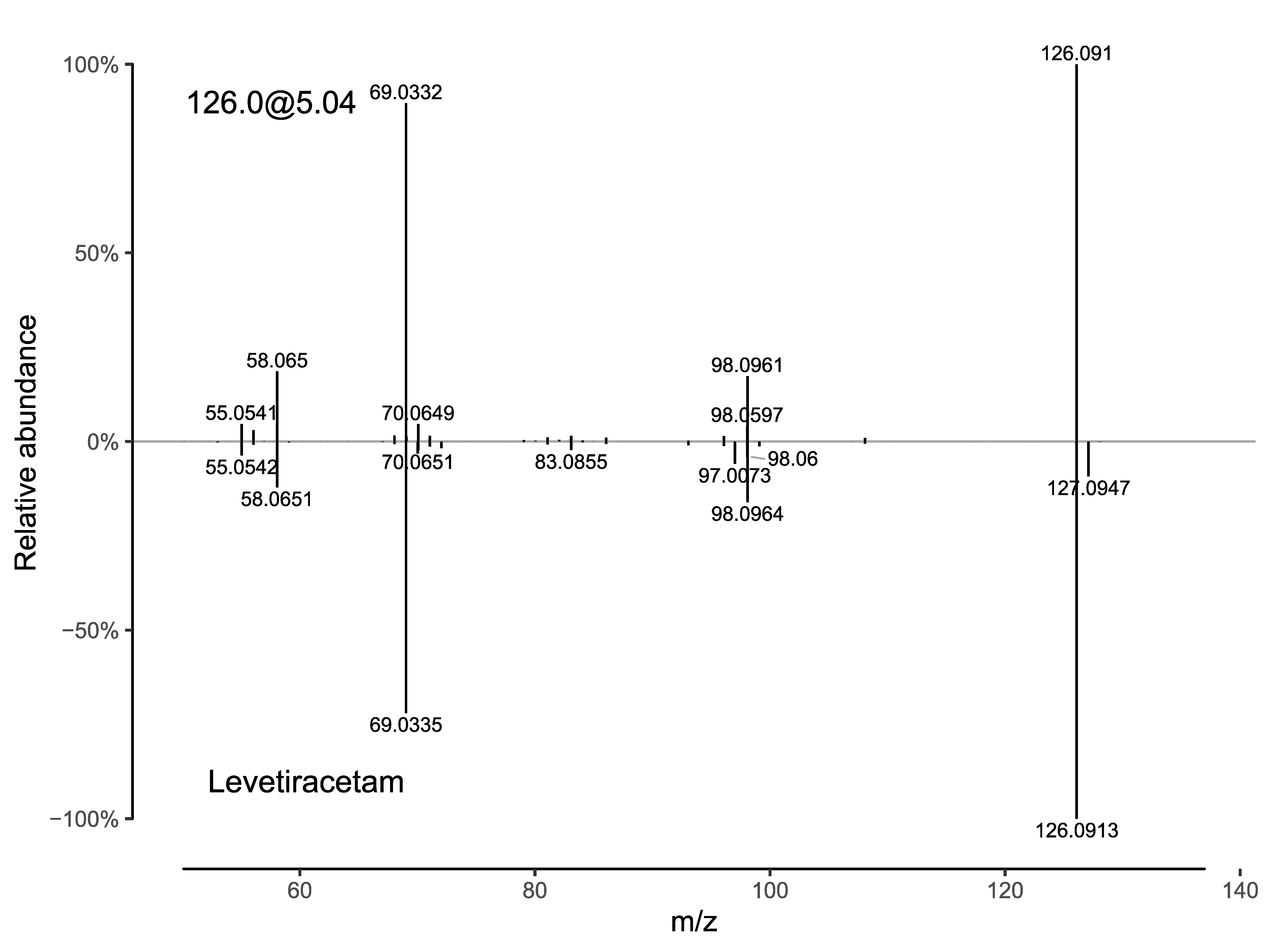


**Figure S7:** MS2 spectrum of feature 126.0@5.04 (top) compared to library MS2 spectrum of levetiracetam (bottom). The 171 m/z molecular ion of levetiracteam was not detected in the library spectrum.

As expected, samples containing 126.0@5.04 also contained a co-eluting feature with the same MS2 spectrum **(Figure S8)**. However, this feature had a molecular ion mass of 172.0966 m/z and a predicted formula of C_8_H_13_NO_3_. This matches a metabolite of levetiracetam previously observed (1) during *in vivo* pharmacokinetic studies (**Figure S9**).


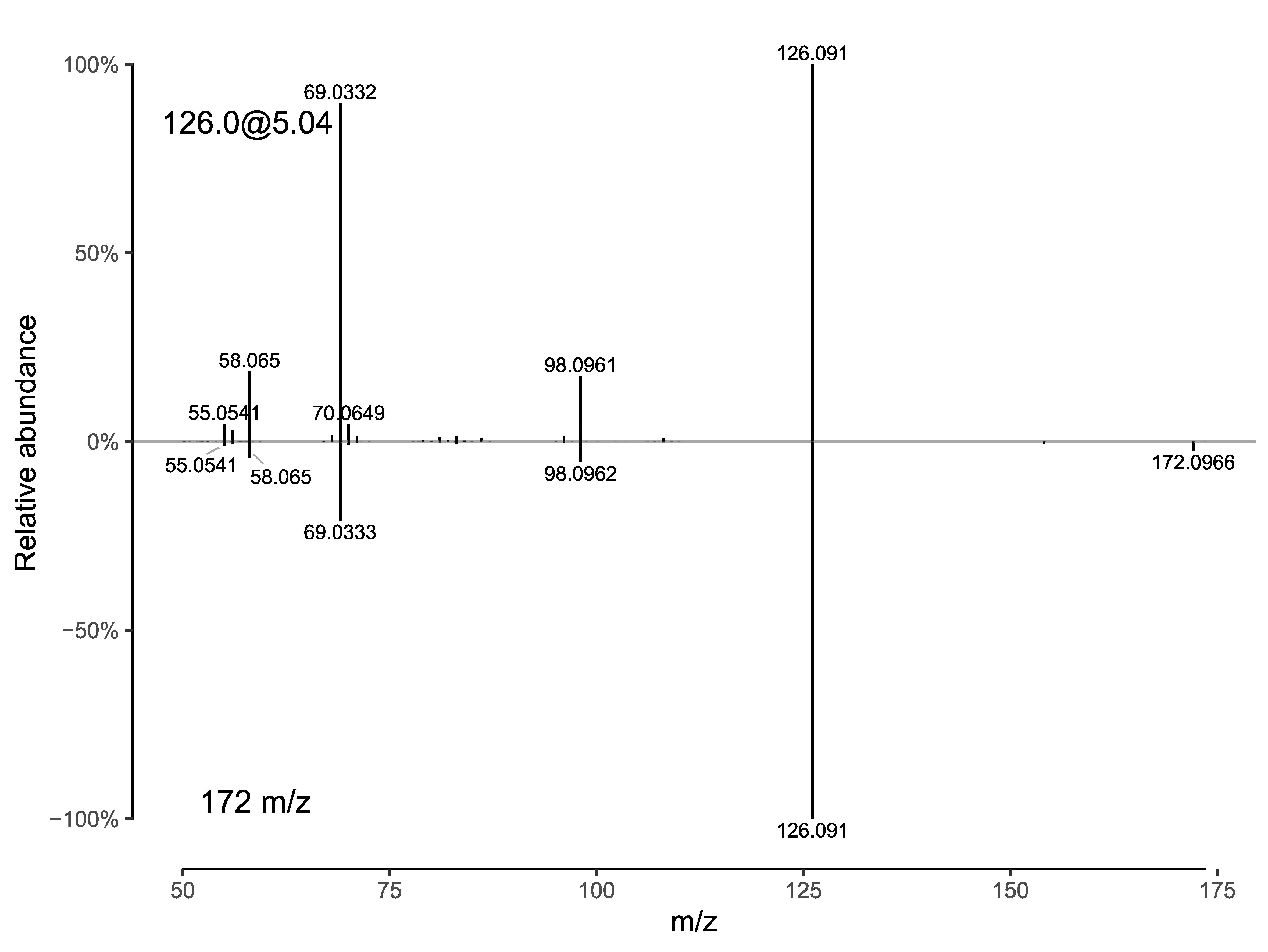


**Figure S8:** MS2 spectrum of 126.0@5.04 (top) compared to MS2 spectrum of the coeluting 172 m/z feature (bottom).


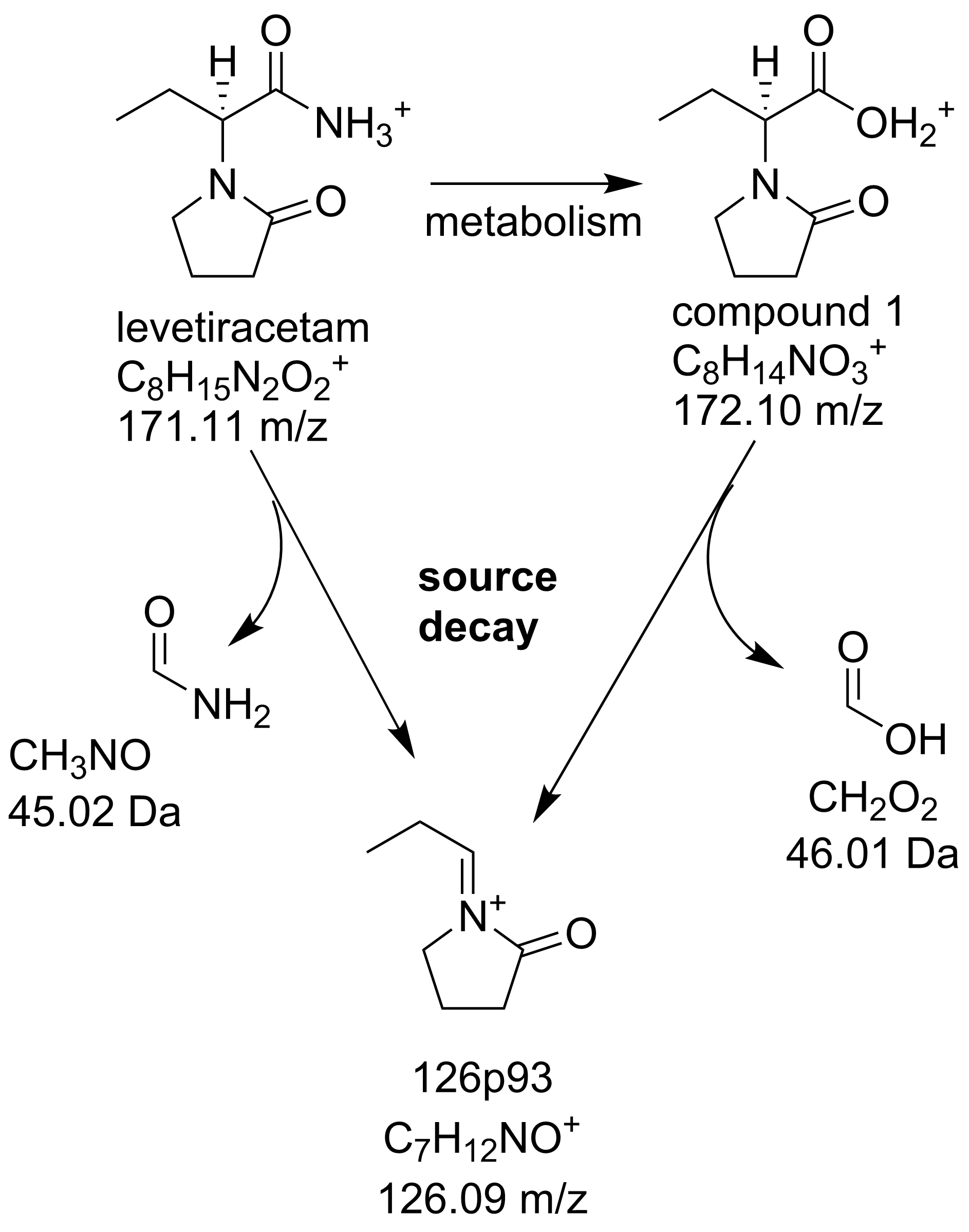


**Figure S9:** Putative structures of 126.0@5.04 and its two source decay precursors. Benedetti *et al.* (1) report *in vivo* conversion of levetiracetam to compound 1. Putative source decay mechanisms produce 126.0@5.04 from either precursor.

The XIC of 126 m/z contains two peaks (**Figure S10**): the 126.0@5.04 feature at 4.06 minutes, and another at 2.59 minutes. This earlier peak has an identical MS2 spectrum to the 126.0@5.04 feature. The 172 m/z putative levetiracetam metabolite elutes only at 4.06 minutes, but a feature with mass 171 m/z coelutes with the 2.59-minute peak. This feature has a predicted formula of C_8_H_14_N_2_O_2_, which matches the formula of levetiracetam. We surveyed several patient samples and found that all four features only occurred together, never separately. Taken together, this evidence strongly suggests that feature 126.0@5.04 is a metabolite of levetiracetam.


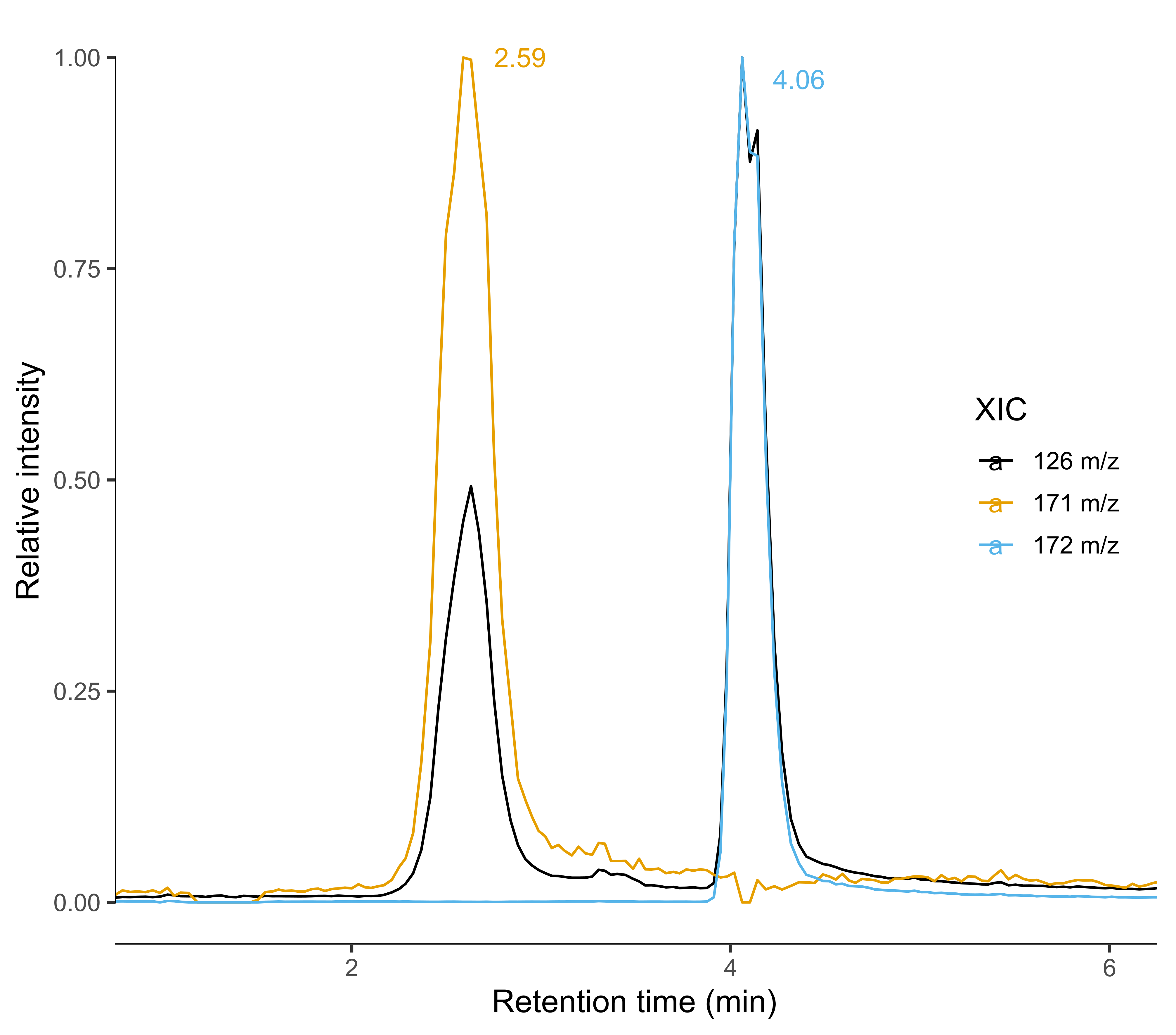


**Figure S10:** Extracted-ion chromatograms of 126.0@5.04 and related features in a single patient sample. Intensities are scaled to the global maximum intensity for each trace.

## Feature 177.1@10.56

The exact mass and isotopologue pattern of feature 177.1@10.56 suggest that its neutral formula is C_12_H_16_O. The presence of a prominent tropylium ion (C_7_H_7_^+^; 91.054 m/z) in its MS2 spectrum indicates that 177.1@10.56 contains a carbon-substituted benzene ring. These substitutions must contain 6 carbons and 1 oxygen. The MS2 spectrum of 177.1@10.56 exhibited partial similarity to the spectrum of a 1-phenyl-2-hexanone reference standard (**Figure S11A**), including the tropylium ion. This suggests that 177.1@10.56 may be structurally similar to 1-phenyl-2-hexanone. The 131.0850 m/z MS2 fragment ion also has a neutral loss of C6H6 (consistent with benzene) in its MS3 spectrum (**Figure S11B**).

**A.**


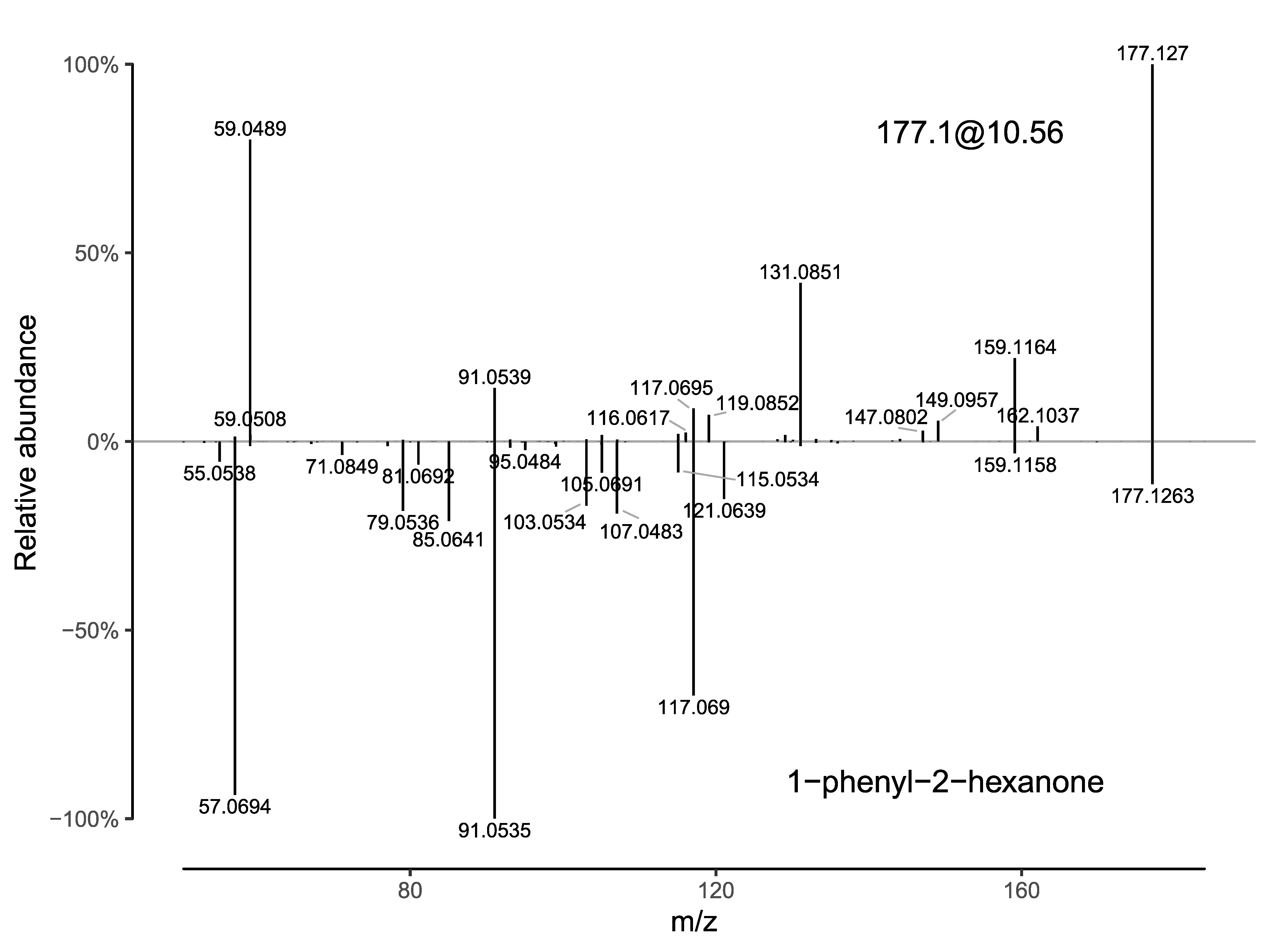


**B.**


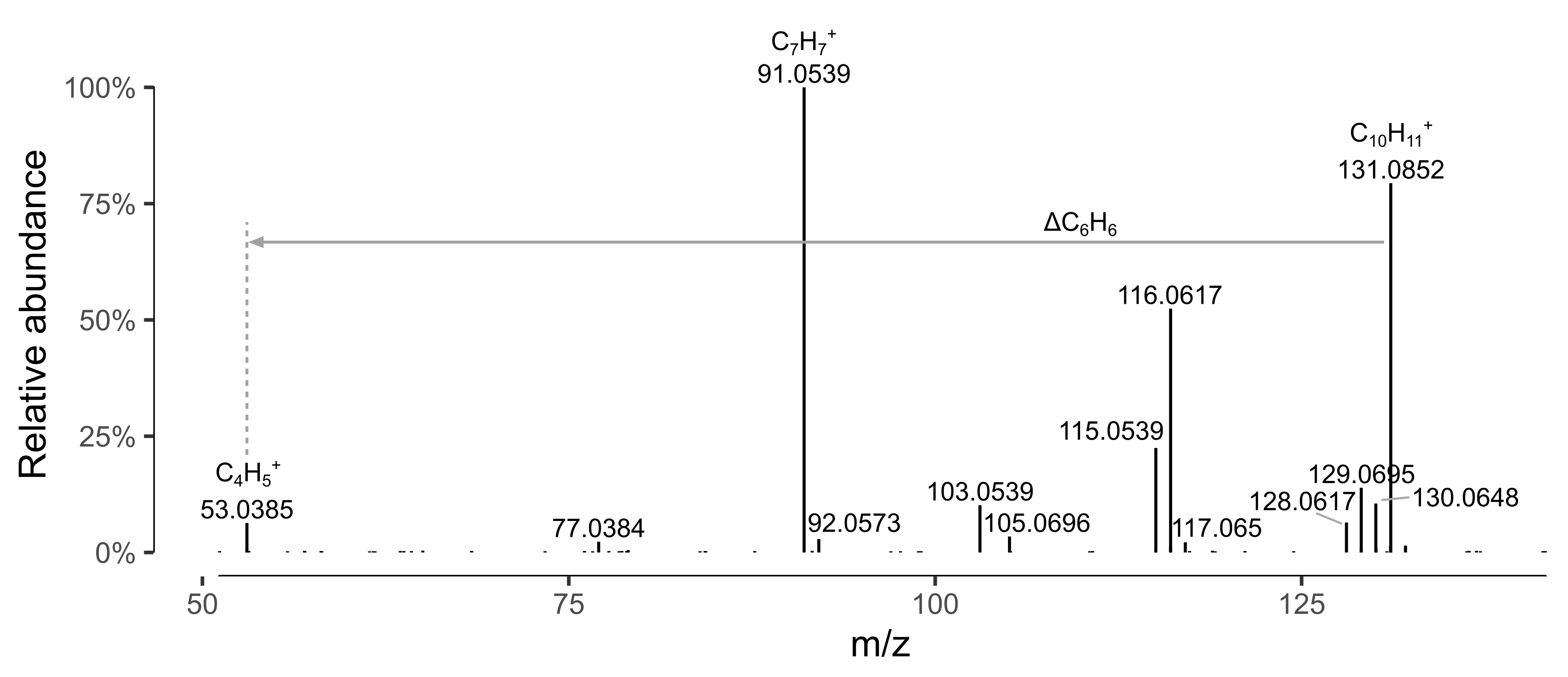


**Figure S11:** (**A**) Comparison of MS2 spectra from 177.1@10.56 (top) and an authentic 1-phenyl-2-hexanone standard (bottom). The fragment ion at 91.0539 m/z matches the tropylium ion, a commonly observed fragmentation product of carbon-substituted benzene rings. (**B**) The MS3 spectrum of MS2 fragment 131.0852 m/z contains a C_6_H_6_ neutral loss consistent with the presence of a benzene ring in this fragment. The tropylium ion is also prominent in this spectrum, further supporting presence of a carbon-substituted benzene.

## Feature 318.9@20.08

Based on the molecular ion mass and isotopologue patterns, the predicted molecular formula is C_18_H_22_O_5_. Spectral similarity matching against the mzCloud spectral library suggested a relationship to enterodiol (C_18_H_22_O_4_, one oxygen less). Comparison of MS2 spectra from 318.9@20.08 and an authentic enterodiol standard confirmed that all enterodiol fragments except 267.1380 m/z (C_18_H_18_O_2_) match a 318.9@20.08 fragment (**Figure S12**). The 267 m/z fragment represents a neutral loss of 2 H_2_O fragments (36.0207 Da), a neutral loss also found in the 318.9@20.08 spectrum (283.1329 m/z, **Figure S13**). In addition, the MS3 spectrum of MS2 fragment 133.0648 m/z is very similar in both feature 318.9@20.08 and enterodiol, further suggesting that 318.9@20.08 is structurally similar to enterodiol (**Figure S14**). These spectral similarities suggest that 318.9@20.08 is likely related to enterodiol and may be an isomer of hydroxyenterodiol.


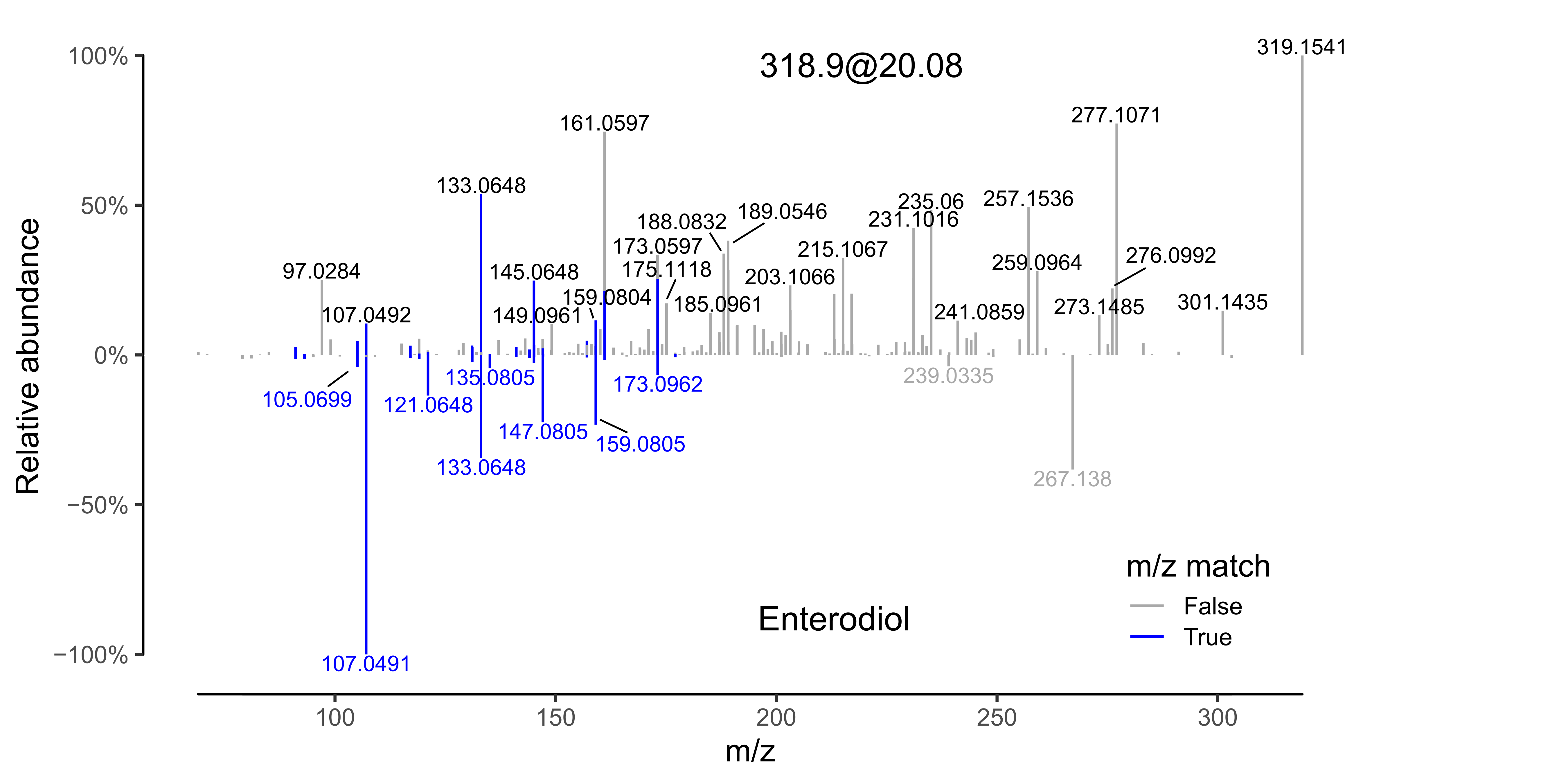


**Figure S12:** Comparison of MS2 spectra from feature 318.9@20.08 and an authentic enterodiol standard. Enterodiol fragments with matching fragments (<25 ppm difference) in the 318.9@20.08 spectrum are marked with blue, while non-matches are marked in grey.


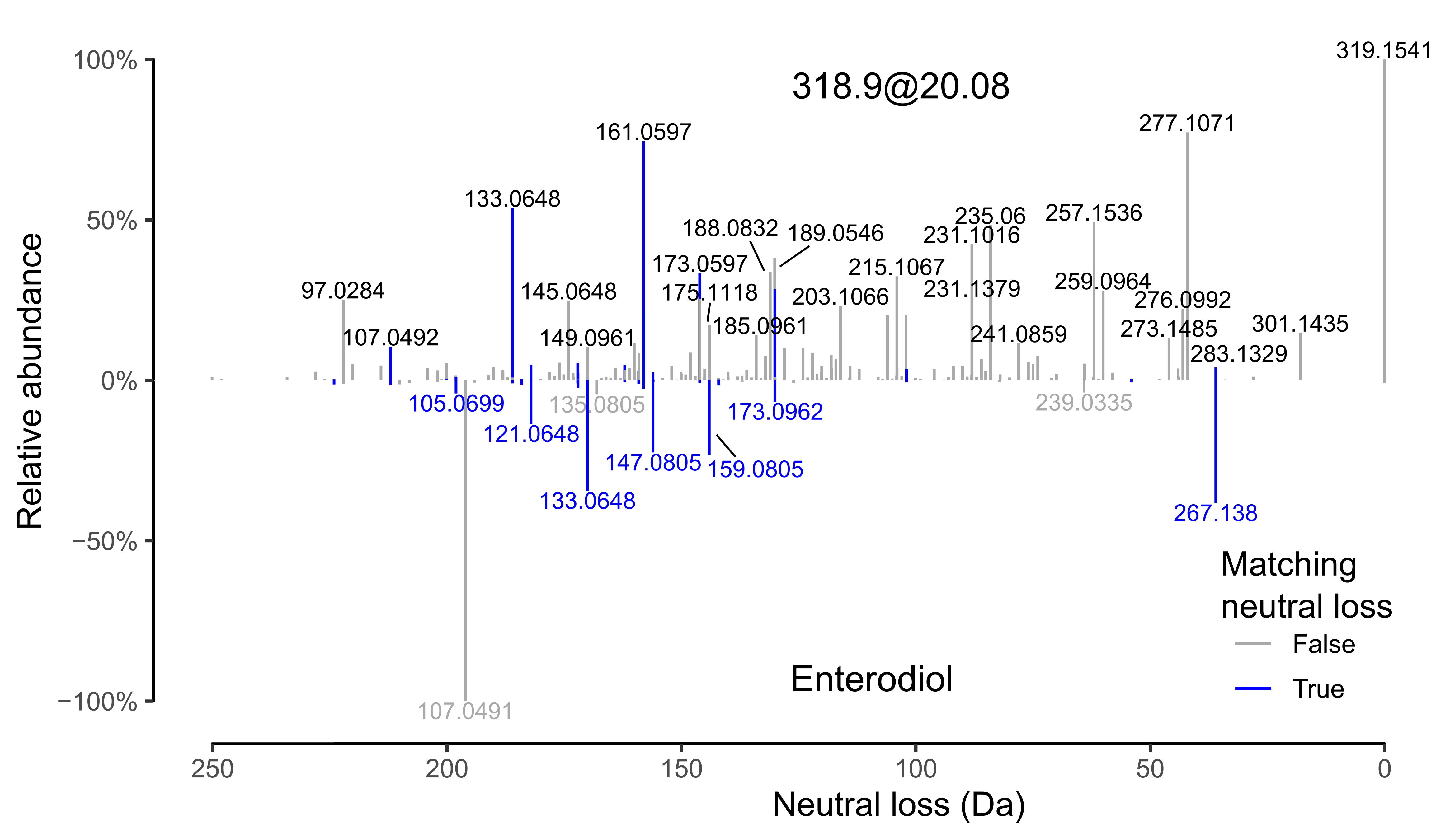


**Figure S13:** Comparison of neutral losses in the MS2 spectra from feature 318.9@20.08 and an authentic enterodiol standard. Enterodiol fragments with matching neutral losses (<25 ppm difference) in the 318.9@20.08 spectrum are marked with blue, while non-matches are marked in grey. Labels indicate absolute m/z ratios of fragments.


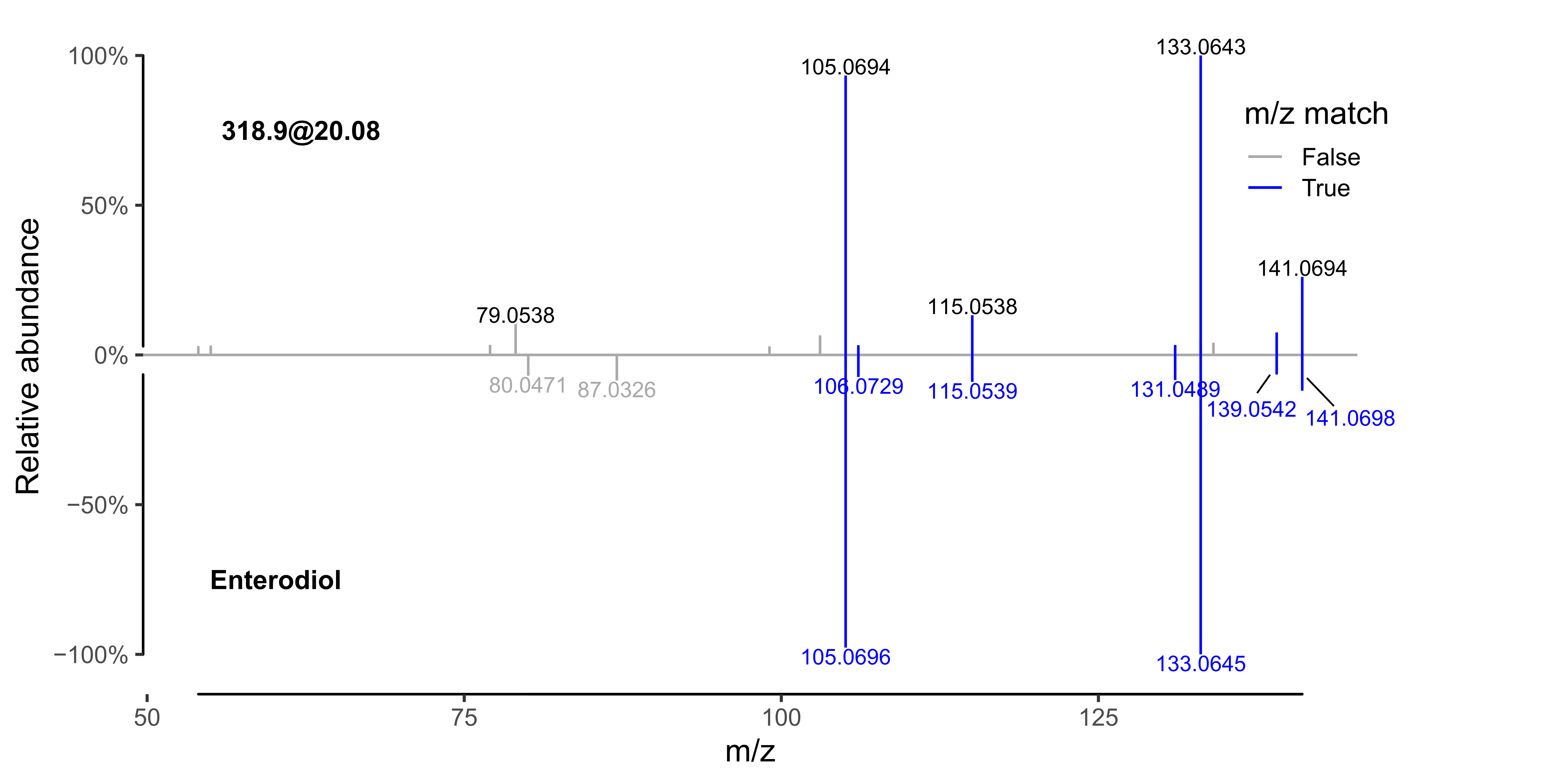


**Figure S14:** Comparison of MS3 spectra of MS2 fragment 133.0648 m/z from feature 318.9@20.08 and an authentic enterodiol standard. MS3 fragments with matching fragments (<25 ppm difference) in both spectra are marked with blue, while non-matches are marked in grey.

# Supplemental model feature selection methods

To estimate the ability of metabolomic and/or clinical data to correctly classify severe outcomes and to minimize overfitting, we performed the initial feature selection process with 5-fold cross-validation train/test splits of the discovery cohort, repeated 20 times with different random seeds, for each combination of metabolomic data and clinical metadata. For the initial genetic algorithm step, we randomly selected an initial population of 100 subsets with 70 metabolites each, with each metabolite’s selection probability weighted by the sum of the differential compositional variation scores of each logratio including that metabolite (2, 3). Metabolite subset fitness was defined as the average out-of-bag AUC of random forest models trained on all pairwise logratios between the metabolites selected (4). Standard elitism, uniform crossover, and point mutation operations were allowed for 50 generations (5). After a subset of metabolites was identified using the genetic algorithm approach, differential compositional variation scores were again used to select subsets of logratios of these metabolites for model training through multi-stage feature selection using a combination of network-based, recursive elimination, and penalized regression techniques (2). This broad approach was used to improve model performance and improve interpretability by eliminating uninformative logratios. Clinical metadata features were optionally selected by applying the Boruta R package to individual training partitions (6). Clinical metadata features were retained if they were selected in 8 of 10 repeats of the Boruta algorithm. The resulting clinical metadata subset was then appended to the selected metabolite subset. After metabolite and/or clinical feature selection, the XGBoost R package was used to train tree booster models using the selected features (7). XGBoost hyperparameters (eta, max depth, min child weight, gamma, subsample, column sample by tree, lambda and alpha) were optimized using coordinate descent. Model AUCs were calculated using the training split’s corresponding test split.

After choosing to proceed with a model combining 70 positive mode metabolomic features and clinical metadata (see main text and **Figure S15** for details), we selected a small representative set of metabolites and clinical attributes by repeating the feature selection process 50 times with positive mode data plus clinical attributes and a target model size of 70 features. We aggregated the variable importance scores for each selected variable across all models trained and, representing each subset as a binary vector, we selected the most representative subset, defined as the subset with minimum total pairwise Hamming distances (**Figure S22**). This most representative subset contained 24 metabolites, which were selected for targeted metabolomic analysis.


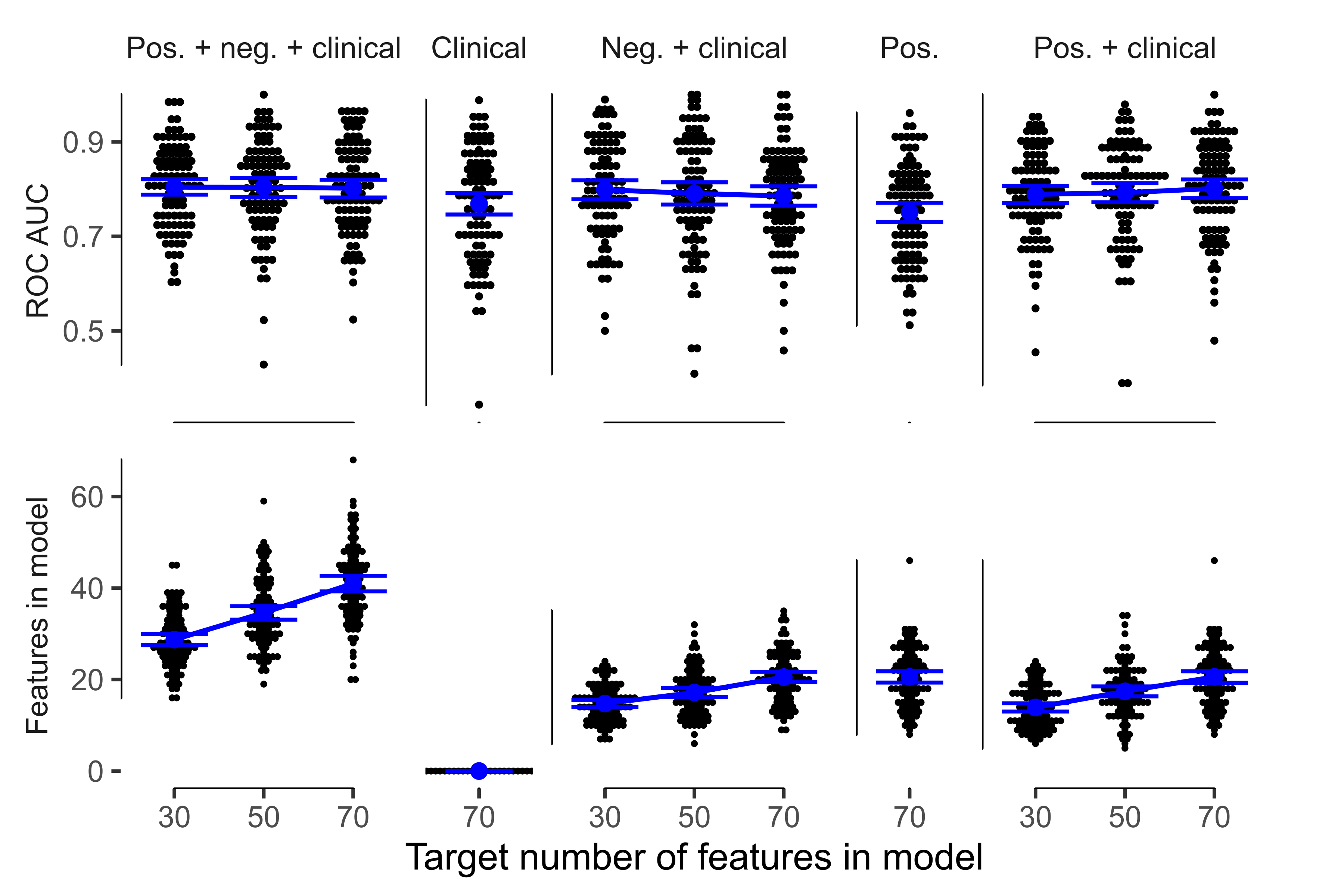


**Figure S15** Cross-validated model performance on the untargeted LC-MS data for several feature number targets. Models were constructed using positive- or negative-mode LC-MS data, optionally including clinical characteristics as well. Error bars (blue) indicate means and bootstrapped 95% confidence intervals.

**
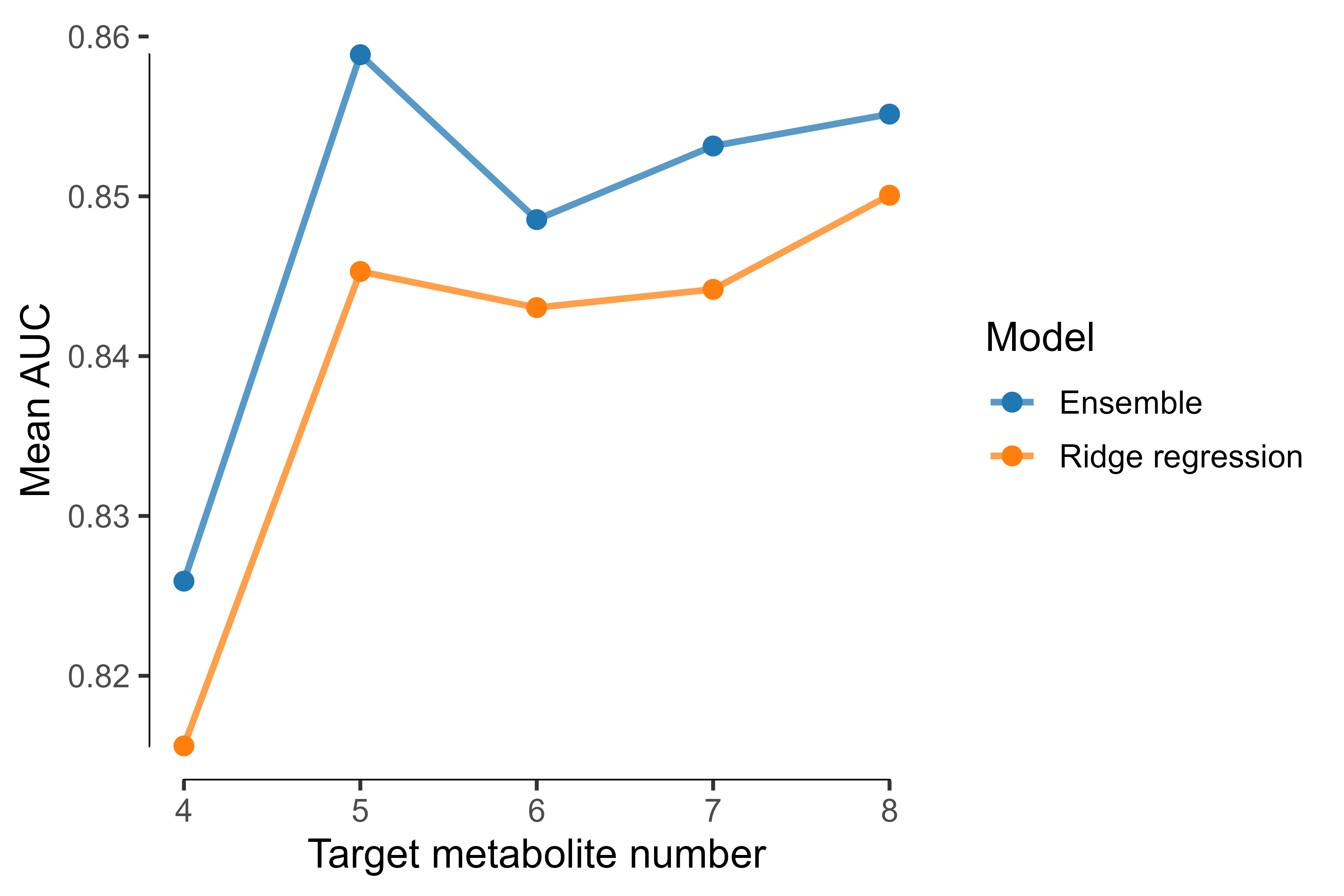
**

**Figure S16:** Estimated model performance on the discovery cohort averaged across nested 2-fold cross validation repeated 5 times. Ensemble models (blue) and ridge-regularized logistic regression models (orange) exhibit similar performance trends, with a peak at 5 features.

**
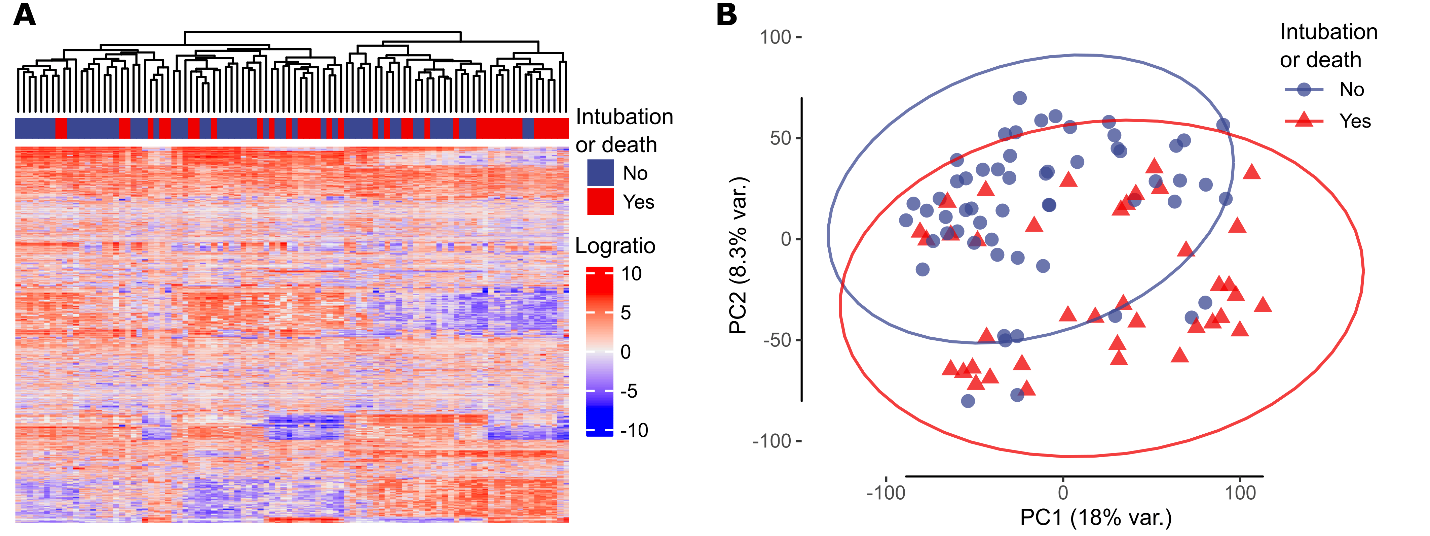
**

**Figure S17. (A)** Heatmap of all pairwise logratios between features detected by untargeted LC-MS. Pairwise logratios were calculated separately for features detected in negative- and positive-ion modes. Red and blue labels above heatmap denote samples with severe or non-severe outcomes, respectively. **(B)** PCA of pairwise feature logratios shows partial separation between patient outcomes.

**
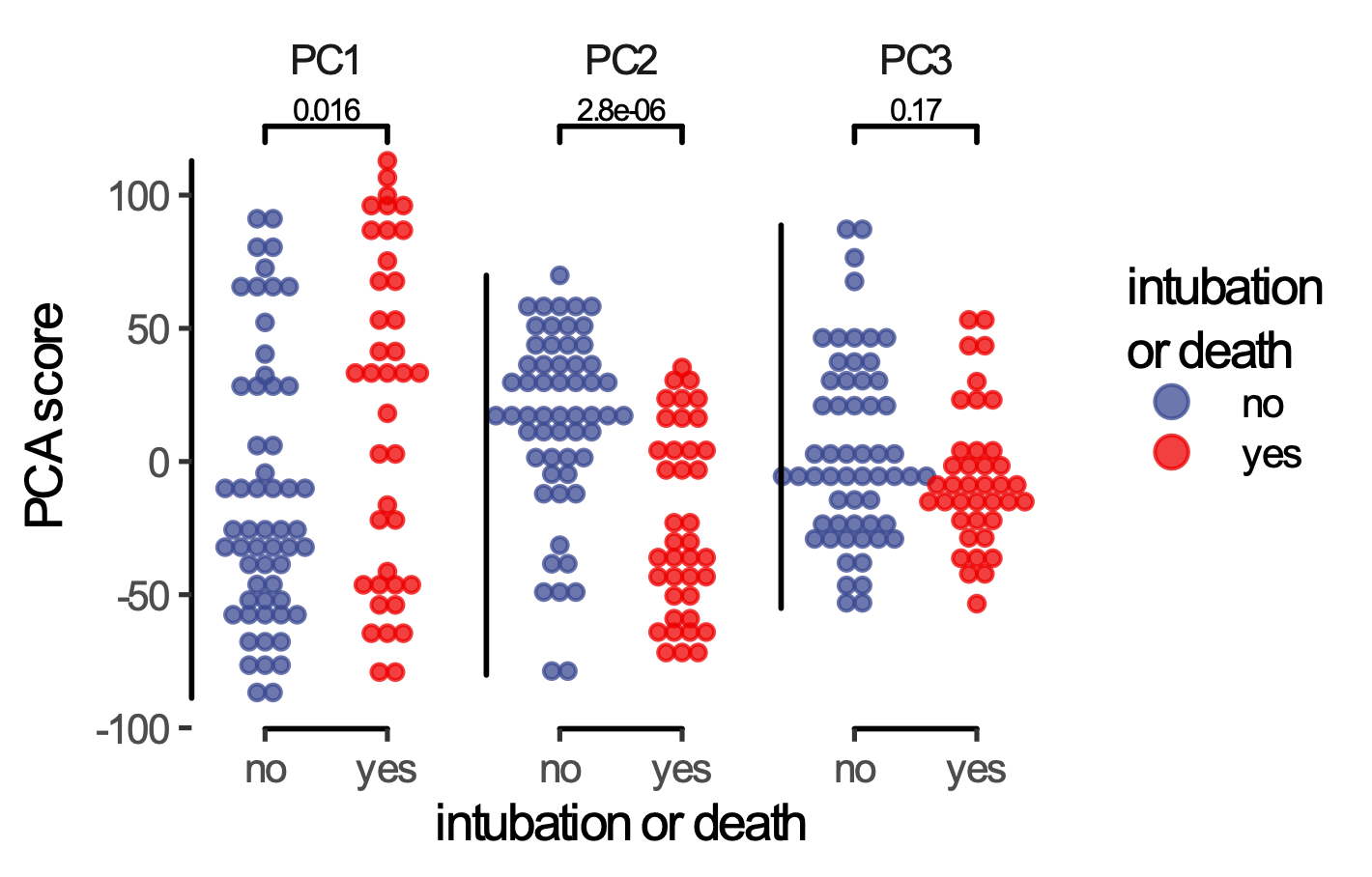
**

**Figure S18:** Comparison of PCA scores on components 1-3 between patients with severe and non-severe outcomes. Scores differ based on outcome in PC1 and PC3 but not PC2 (two-tailed Wilcoxon signed-rank test).

**
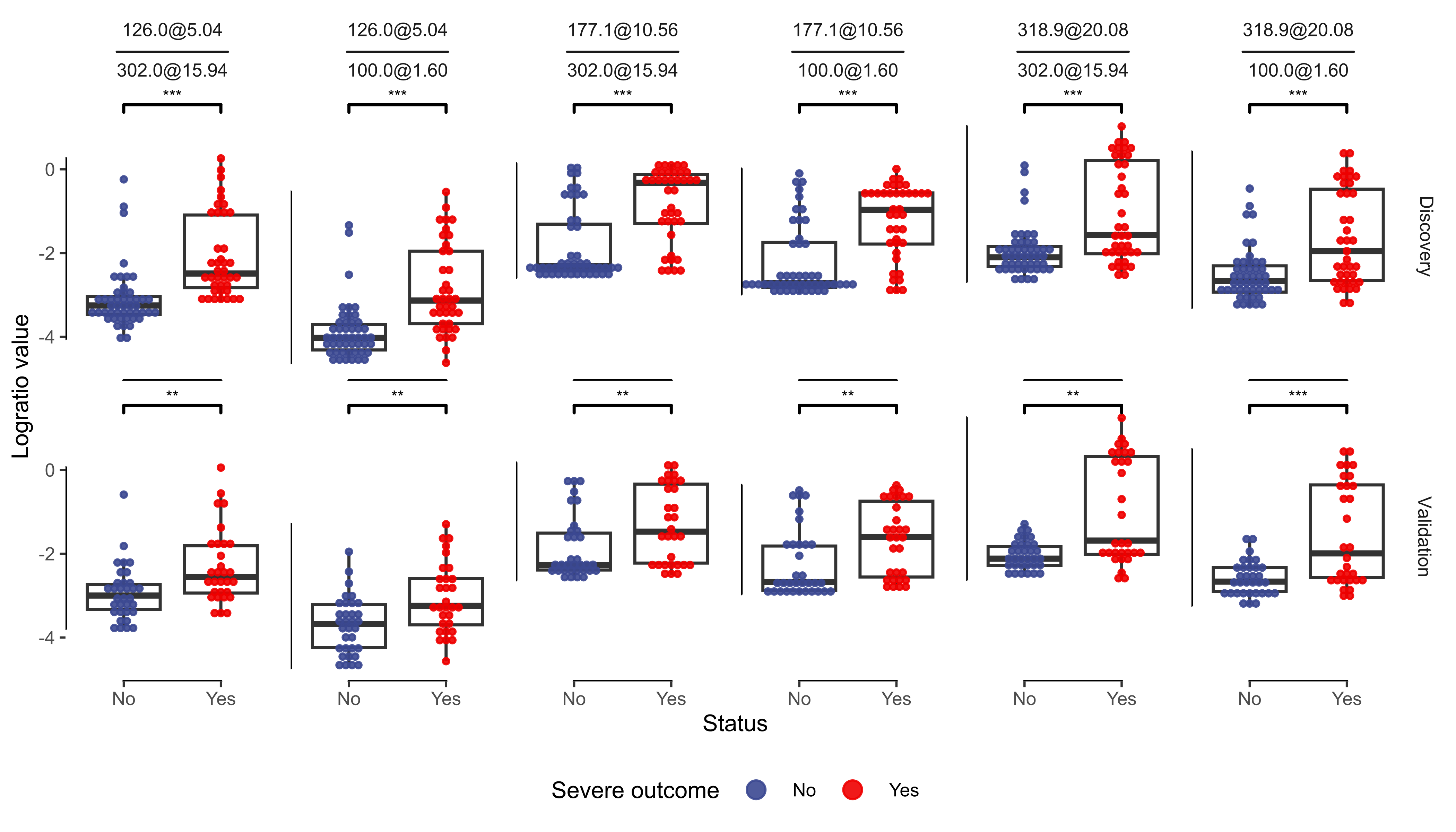
**

**Figure S19:** Metabolite logratios included in the final model are all significantly elevated in patients with severe outcomes in both the discovery and validation cohorts. P-values were calculated using the Wilcoxon rank sum test (** p<0.01, *** p<0.001).

**Table S3.** Correlative model performance using the standard 50% probability cutoff.

|  | **Discovery** | **Validation** |
| --- | --- | --- |
| Accuracy | 81.6% | 74.6% |
| Negative predictive value | 82.9% | 79.2% |
| Positive predictive value | 80.8% | 71.8% |
| Sensitivity | 87.5% | 84.9% |
| Specificity | 74.4% | 63.3% |

**A.**


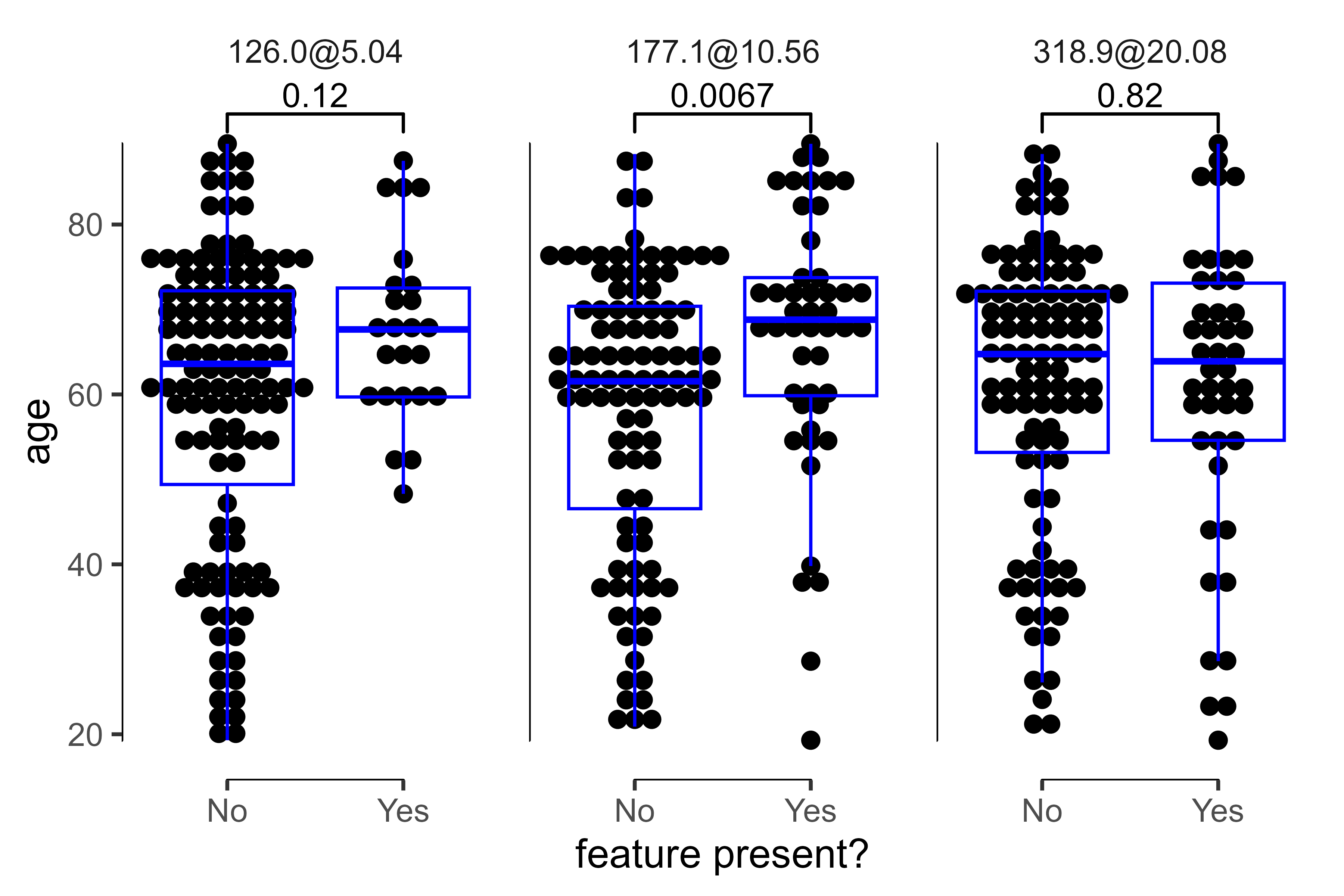


**B.**


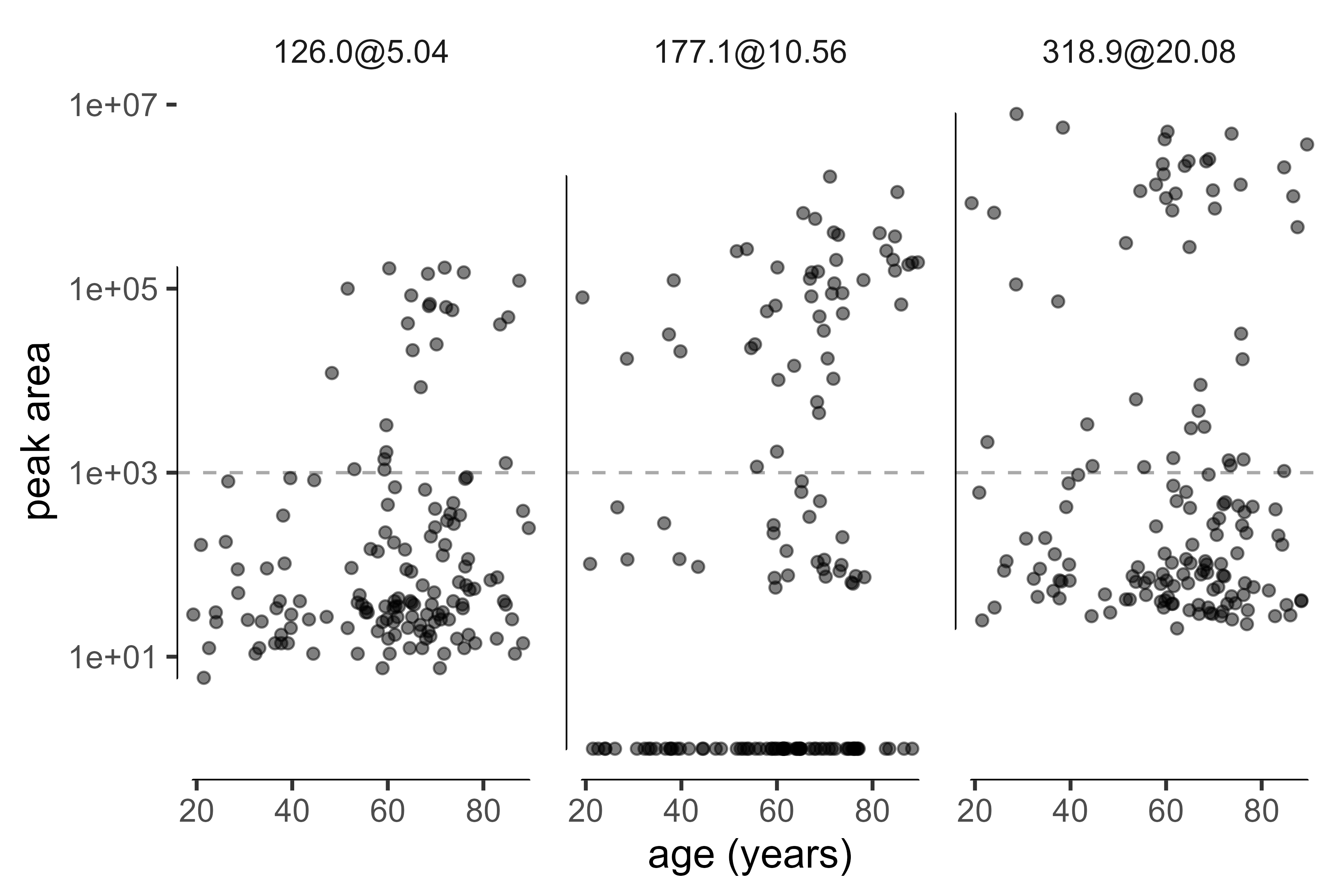


**Figure S20:** Out of the three metabolites correlated with severe outcomes, only feature 177.1@10.56 exhibits a relationship with patient age. **(A)** Patients with 177.1@10.56 detected in their urine (area > 1000) are significantly older than patients without it (p<0.01, Wilcoxon rank-sum test). **(B)** Relationship between patient age and metabolite peak areas, showing correlation between 177.1@10.56 levels and patient age.


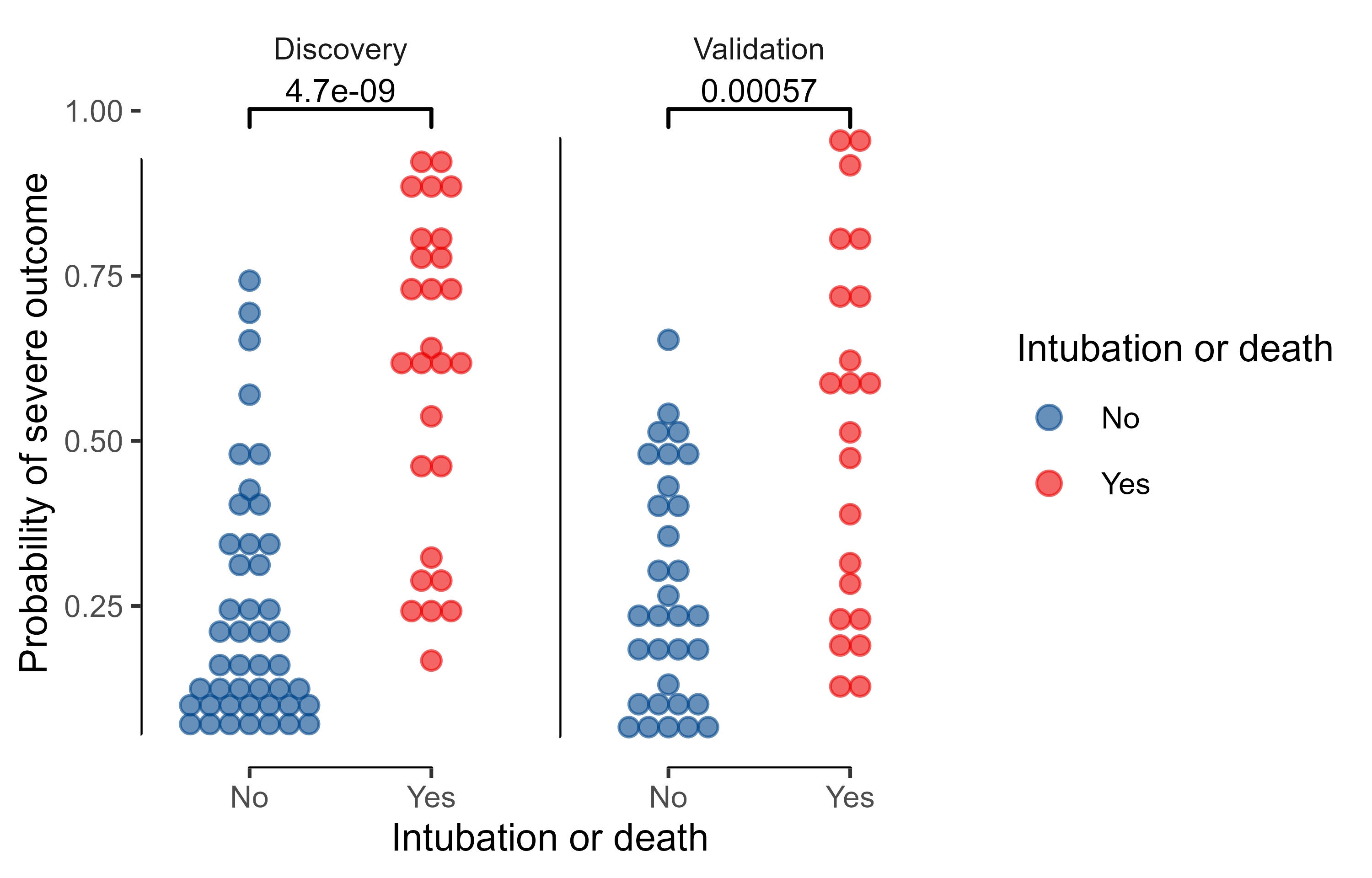


**Figure S21:** Final logistic regression model remains correlative even when levetiracetam-positive patients are held out. Model probabilities of severe outcome are shown for only patients with levetiracetam (feature 126.0@5.04) levels below the limit of detection (1000 counts). The model correctly assigns significantly higher probabilities of severe outcome to patients who were intubated or died (p<0.001, Wilcoxon rank sum test).

# Metabolite signature robustness

The machine learning processes we describe in this work exhibit some intrinsic variation due to randomness in variable selection and cross-validation fold assignment. This can be readily observed in the genetic algorithm results (**Figure S22**), where a different choice of random seed would result in somewhat different subsets of LC-MS features being selected. Although 3 of 5 members of the final biomarker signature were selected in all 50 seeds, features 126.0@5.04 and 177.1@10.56 were only selected 36 and 34 times, respectively.


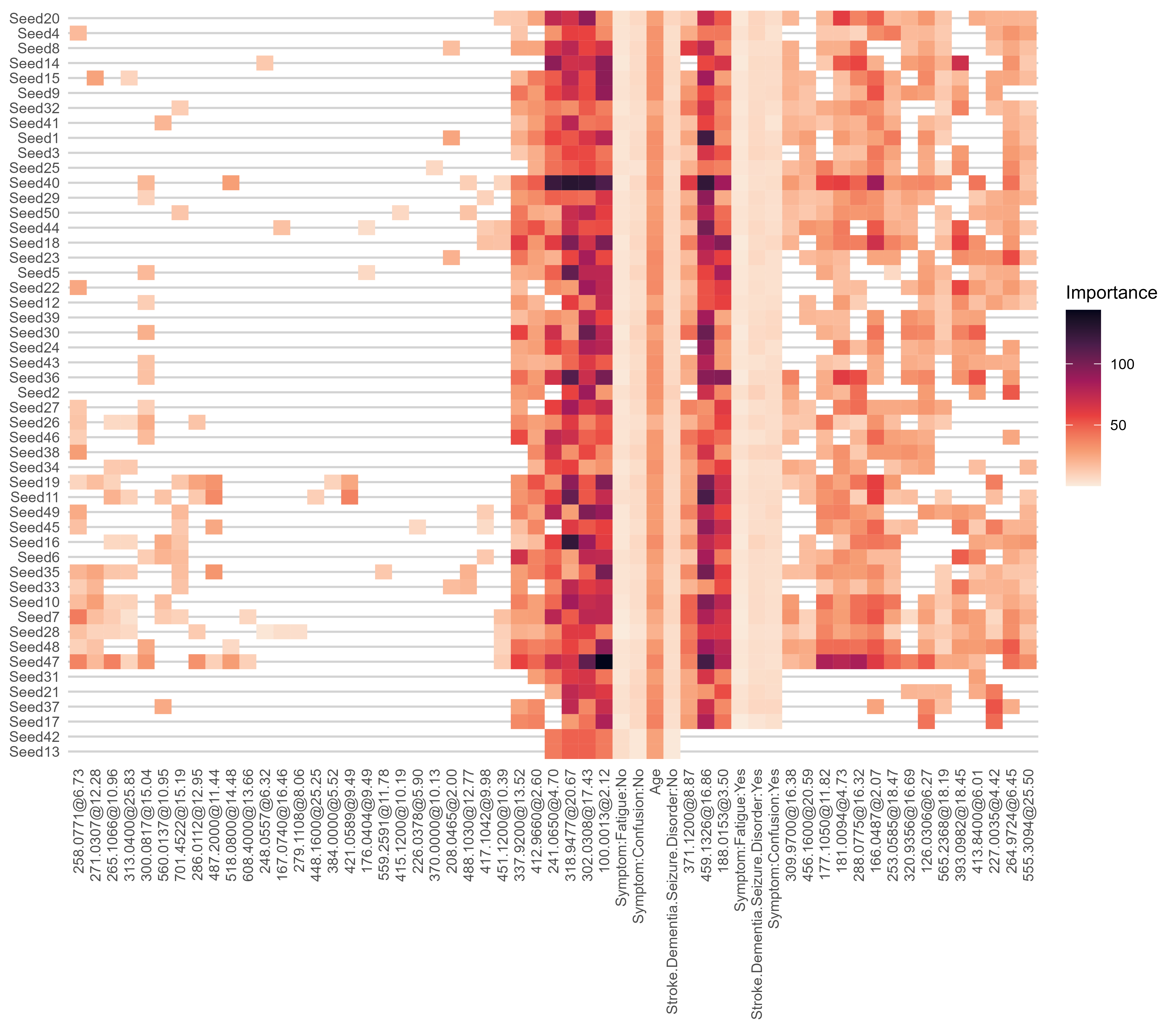


**Figure S22:** Putative correlative metabolite subsets selected by bounded feature selection.

To explore whether the COVID-19 severity associated metabolite signature is robust to choices made during the machine learning process, we performed *post hoc* analyses with different model settings. The metabolite signature presented in this work was chosen using an arbitrarily selected random number generator seed, so we repeated the selection process using 25 randomly-selected seeds. In addition, we originally chose to use the model size that yielded the highest average AUC during inner cross-validation. Here we test another approach, used in the glmnet R package, which selects the smallest model size with a cross-validated mean AUC within one standard error of the highest mean AUC (8). This method typically selects fewer features. Finally, although it might result in a certain amount of information leakage between training and testing sets, we also test the effect of calculating a shared multiplicative imputation factor for zero imputation across the whole discovery cohort during cross-validation and across both discovery and validation cohorts when building the final model.


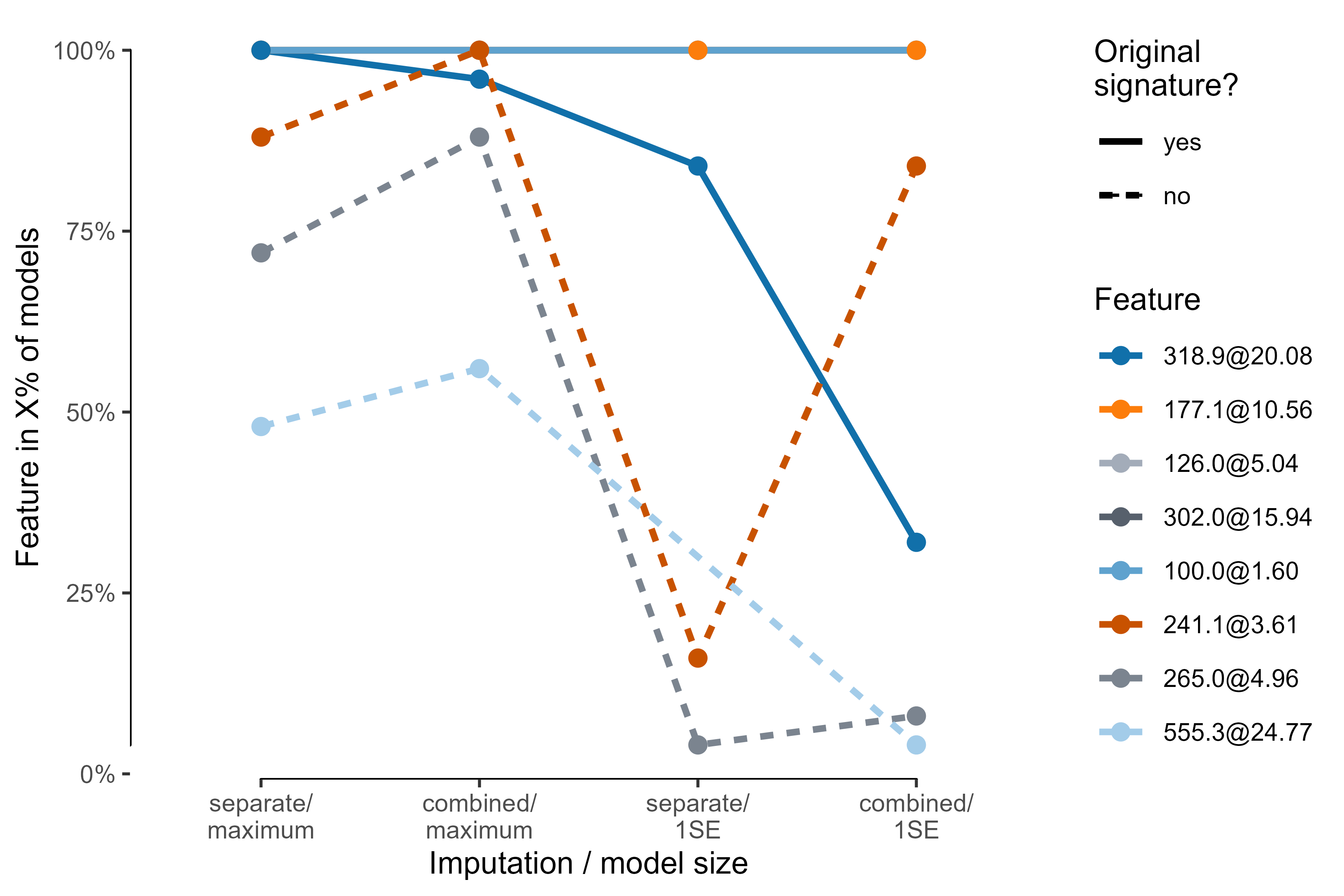


**Figure S23:** Final DiCoVar model fitting selects a small subset of features when trained using different parameters across 25 separate modeling runs. The original metabolite signature was found using separate imputation and maximum AUC. Features 100.0@1.60, 126.0@5.04, and 177.1@10.56 are selected in every model. Features not in the original metabolite signature are marked with dashed lines.

Every model generated using this process contained 4 of the 5 metabolites in our main metabolic signature (**Figure S23**). In some models, metabolic signature feature 318.9@20.08 is replaced by one or more other features, frequently feature 241.1@3.61. However, models fit using the original parameters (separate imputation and maximum AUC), always include 318.9@20.08. Notably, models across all parameter combinations only select from a subset of 8 features out of the 24 available. This suggests that a consistent subset of features are strongly correlated with severe COVID-19 and that the original metabolic signature captures the 5 most important of these.

**
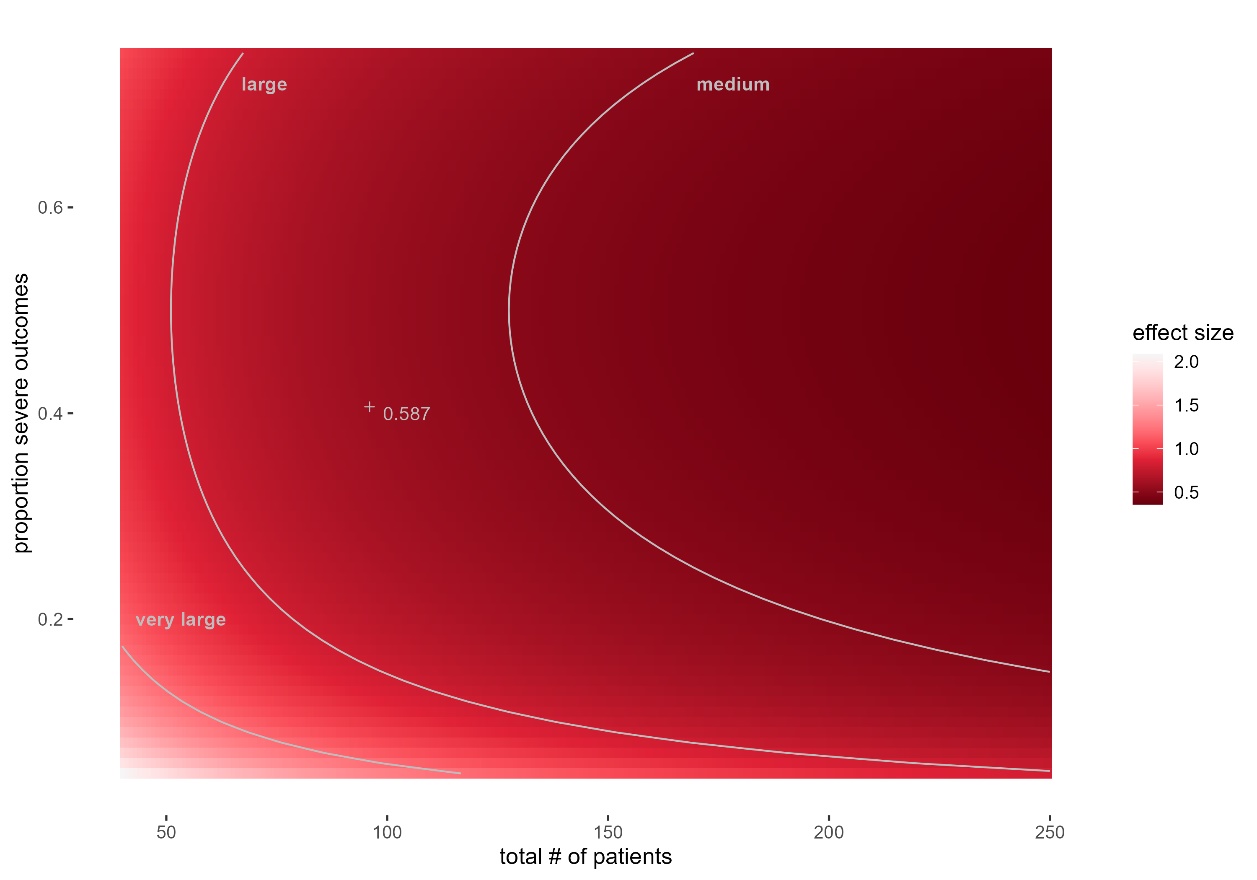
**

**Figure S24:** Assuming that prognostic metabolites will have approximately log-normal distributions with different means depending on outcome, we predicted minimum detectable effect sizes at the 0.05 significance level with power of 0.8 (Cohen 1988). The results are plotted as a heatmap with the standard effect sizes superimposed. By this calculation, the discovery cohort in the current study could detect effect sizes above 0.587. The three numerator metabolites found in this study have observed effect sizes of 0.52, 0.53, and 1.01, suggesting that this calculation is at least roughly correct.





**Figure S25:** Performance of the final DiCoVar model (DCV) in the discovery and validation cohorts compared to models including clinical characteristics and to models excluding metabotype 3 (Metabotypes 1/2). ROC AUC comparisons and 95% confidence intervals were computed by bootstrapping and corrected for multiple comparisons using Bonferroni’s method (6 comparisons within cohort, 3 comparisons between cohorts). No comparisons were significant (p>0.05). Vertical dashed line indicates 50% ROC AUC.

# References

1. Strolin Benedetti M, Whomsley R, Nicolas JM, Young C, Baltes E. 2003. Pharmacokinetics and metabolism of 14C-levetiracetam, a new antiepileptic agent, in healthy volunteers. Eur J Clin Pharmacol 59:621-30.

2. Hinton AL, Mucha PJ. 2022. A Simultaneous Feature Selection and Compositional Association Test for Detecting Sparse Associations in High-Dimensional Metagenomic Data. Frontiers in Microbiology 13:837396.

3. Hinton AL, Mucha PJ. 2021. Differential Compositional Variation Feature Selection: A Machine Learning Framework with Log Ratios for Compositional Metagenomic Data doi:10.1101/2021.12.08.471758, p 2021.12.08.471758.

4. Breiman L. 2001. Random Forests. Machine Learning 45:5-32.

5. Mitchell M. 1998. An Introduction to Genetic Algorithms doi:10.7551/mitpress/3927.001.0001. The MIT Press.

6. Kursa MB, Rudnicki WR. 2010. Feature Selection with the Boruta Package. Journal of Statistical Software 36:1-13.

7. Chen T, Guestrin C. 2016. XGBoost: A Scalable Tree Boosting System, abstr Proceedings of the 22nd ACM SIGKDD International Conference on Knowledge Discovery and Data Mining, San Francisco, California, USA, Association for Computing Machinery,

8. Friedman JH, Hastie T, Tibshirani R. 2010. Regularization Paths for Generalized Linear Models via Coordinate Descent. Journal of Statistical Software 33:1 - 22.
